# Uncovering Diaphragm Cramp in SIDS and Other Sudden Unexpected Deaths

**DOI:** 10.3390/diagnostics14202324

**Published:** 2024-10-18

**Authors:** Dov Jordan Gebien, Michael Eisenhut

**Affiliations:** 1 Independent Researcher, Toronto, ON M2K 1G4, Canada; 2 Luton & Dunstable University Hospital, Lewsey Road, Luton LU4 0DZ, UK; michael_eisenhut@yahoo.com

**Keywords:** apnea, contraction band necrosis, DCC, diaphragm cramp, diaphragm fatigue, diaphragm spasm, myopathy, respiratory arrest, SIDS, sudden unexpected deaths

## Abstract

The diaphragm is the primary muscle of respiration. Here, we disclose a fascinating patient’s perspective that led, by clinical reasoning alone, to a novel mechanism of spontaneous respiratory arrests termed diaphragm cramp-contracture (DCC). Although the 7-year-old boy survived its paroxysmal nocturnal “bearhug pain apnea” episodes, essentially by breathing out to breathe in, DCC could cause sudden unexpected deaths in children, especially infants. Diaphragm fatigue is central to the DCC hypothesis in SIDS. Most, if not all, SIDS risk factors contribute to it, such as male sex, young infancy, rebreathing, nicotine, overheating and viral infections. A workload surge by a roll to prone position or REM-sleep inactivation of airway dilator or respiratory accessory muscles can trigger pathological diaphragm excitation (e.g., spasms, flutter, cramp). Electromyography studies in preterm infants already show that diaphragm fatigue and sudden temporary failure by transient spasms induce apneas, hypopneas and forced expirations, all leading to hypoxemic episodes. By extension, prolonged spasm as a diaphragm cramp would induce sustained apnea with severe hypoxemia and cardiac arrest if not quickly aborted. This would cause a sudden, rapid, silent death consistent with SIDS. Moreover, a unique airway obstruction could develop where the hypercontracted diaphragm resists terminal inspiratory efforts by the accessory muscles. It would disappear postmortem. SIDS autopsy evidence consistent with DCC includes disrupted myofibers and contraction band necrosis as well as signs of agonal breathing from obstruction. Screening for diaphragm injury from hypoxemia, hyperthermia, viral myositis and excitation include serum CK-MM and skeletal troponin-I. Active excitation could be visualized on ultrasound or fluoroscopy and monitored by respiratory inductive plethysmography or electromyography.

## 1. Introduction

In early 2023 a patient shared a fascinating medical history suggesting the existence of a previously unrecognized mechanism of spontaneous respiratory arrests in children which we termed, novel “diaphragm cramp-contracture” (DCC) [1]. While he survived its repeated paroxysmal nocturnal bearhug pain apneas, DCC could be fatal, especially in infants. After intensive literature review, writing and communications, and despite remaining skeptical throughout, we have come to suspect DCC as a terminal pathologic mechanism in many types of non-arrhythmogenic sudden unexpected deaths. This includes those in infants (SUDI, including SIDS), older children (SUDC), epilepsy (SUDEP) as well as some sudden cardiac deaths (SCD) and even severe abdominal winding injuries. In addition, it appears transient diaphragm spasms in preterm infants contribute to forced expirations, apneas-hypopneas and periodic breathing, all of which are known to cause recurrent hypoxemic episodes that are associated with serious long-term morbidity and mortality (including SIDS and SUDC). The same could induce breath-holding spells in older children as well as sleep-disordered breathing at all ages, including some obstructive and “central” sleep apneas. Those too are associated with major cardiovascular disorders occurring over the long term, such as hypertension, arrhythmias and SCD.

## 2. Discussion

### 2.1. Patient “0”—Caucasian 52-Year-Old Healthy Male

Though an adult at presentation, the case patient shared a harrowing story of painful nocturnal respiratory arrests that sporadically afflicted him throughout his childhood and youth. He felt he came within a breath of losing his life each time. Starting at 7 years old one night, he suddenly awoke gasping (mouth opened involuntarily, unable to inspire) with bilateral rib pain that felt like someone had picked him up from behind in a tight bearhug. The pain was characterized as pleuritic and cramp-like in nature. He did not recall being ill at the time or sharing the bed; however, did have many risk factors overlapping with those identified in SIDS (e.g., household cigarette smoke, cold climate, nocturnal sweating, deep sleeper with tendency to pull blankets over shoulders and head and a history of severe gastroesophageal reflux [2], requiring hiatal hernia repair at 18 months of age). When he tried inspiring forcefully, it was met with more pain and complete resistance to airflow. Realizing it was futile, he experimented—as only a young child can despite the duress of nearing death—learning he could still exhale. Forced inspirations again futile, he felt he then saved his life by exhaling, followed quickly by three short burst (staccato) high-pressure inhalations “like a pilot breathing in a centrifuge” (Supplementary Video S1). Immediately, the pain and obstruction disappeared, and normal breathing resumed. He then went back to sleep as if nothing had happened. With sporadic recurrences over the next ten years, always at night while asleep, he reemployed this breathing technique and even learned to do it in his sleep. He commented, “Over the years I became intricately aware of this thing, fearing the pain and inability to breathe, learning to abort it by recognizing its [prodromal painful rib fasciculations] and quickly taking in a breath before the big pain would kick in [at end-inspiration]” (obviating the need for the rescue breaths). As he grew older, he just assumed this happened to everybody, perhaps minimizing the traumatic events as a psychological defense mechanism. Sadly, he never told anyone until 45 years later. For more details, see Patient’s Perspective in Supplementary Materials.

**Figure 1:**
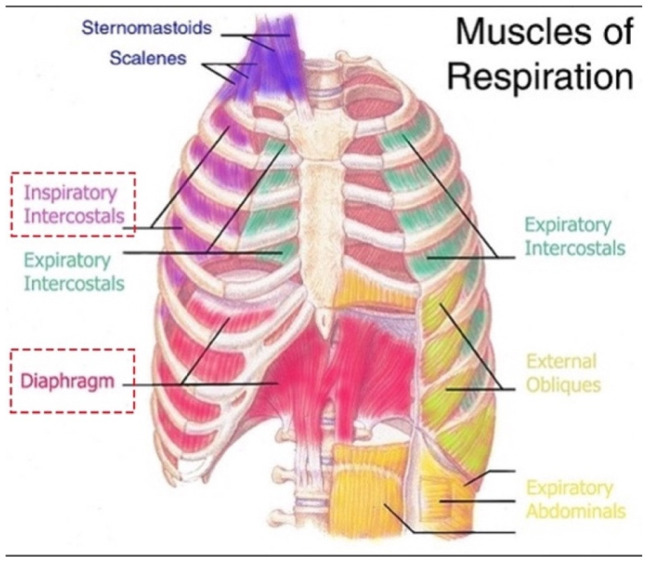
Muscles of respiration. The primary inspiratory muscles are the diaphragm and paired groups of left and right external intercostal muscles (ICMs, lavender). The latter reduce diaphragmatic workload by their bucket handle movements in adults, which basically widen the ribcage. Theoretically, if the bilateral ICMs were to suddenly fail by cramping, apnea would not occur as long as the diaphragm continued functioning (because it is the main inspiratory muscle). Contrarily, however, if the diaphragm fails, apnea and inspiratory arrest could ensue, but only if the ICMs are unable to independently resume ventilation. Therefore, for apnea to occur, both must fail. With permission by Concept2. Unit C8, Crossgate Drive, Queens Drive Industrial Estate, Nottingham NG2 1LW, UK.

Although beyond the scope of this paper, most items in the differential diagnosis (Table S1) were easily excluded using clinical reasoning of key historical features: spontaneous sudden-onset recurrent nocturnal cramp-like bilateral rib (bearhug) pain with complete inspiratory arrest. The most common causes of nocturnal respiratory distress in children, such as panic attacks, sleep paralysis, night terrors, seizures, bronchospasm, laryngospasm and OSA, were ruled out simply because they do not feature intense pain. Similarly, painful conditions of the ribs or chest do not include a 100% airway obstruction. This left the novel diagnosis of bilateral cramps of the diaphragm and intercostal muscles (Figure 1). Alternatively, the entire story could have been fictitious or related to child abuse (both denied). However, the patient’s account was exceptionally detailed and consistent throughout, supporting his authenticity and credibility. Given it is possible a cramp could localize to a region within a muscle body, yet apnea occurred (failure of the entire inspiration movement), it was necessary to introduce the term, contracture. Thus, diaphragm cramp-contracture was adopted. It was hypothesized that loss of diaphragm mechanical pump function by DCC induced a state equivalent to acute bilateral diaphragmatic paralysis (explaining the patient’s inspiratory arrest).

Why this was medically unheard of could relate to extremely high mortality (few survivors). Also, if there are any, they would be preverbal children unable to describe symptoms or older ones, like the case patient, who repressed or were reluctant to share memories. Lastly, it occurs during deep sleep, making recall and differentiation of reality from dreaming less clear. Table A1 provides further speculation as to how DCC might have evaded detection historically.

### 2.2. Evidence for Putative DCC in Medical Literature

Next, the SIDS and SUDC literature bodies were investigated, looking to rule out DCC. The search revealed Poets et al.’s (1999) [3] report on nine preterm infants aged 1–6 months who had succumbed to SIDS and had basic home cardiorespiratory “memory monitor” recordings of their final moments (heart rate and respiratory movements but not airflow or oxygen saturations). Cause of the monitor alarm was bradycardia in seven and apnea in two. Identical to the case patient’s report, terminal gasping and inspiratory arrest had occurred (in seven infants). Gasping duration ranged from 3 s to 11 min. Also, it was ineffective, whereby efforts to inspire were unsuccessful in reestablishing airflow and reversing the bradycardia. This indicated an airway obstruction existed, but to this day, has never been identified other than speculated over laryngospasm or bronchospasm. Furthermore, the authors explained how gasping in mammals occurs when PaO_2_ falls under 5–15 mmHg (normal range in infants is 50–80 mmHg) and is elicited only by hypoxemia, not hypercapnia or acidosis. This suggested severe hypoxemia was present before the monitors had alarmed. As prolonged central apneas were not detected, potential causes were reduced to blunted chemoreceptor responses, hypoventilation and breathing exhaled gases.

**Figure 2:**
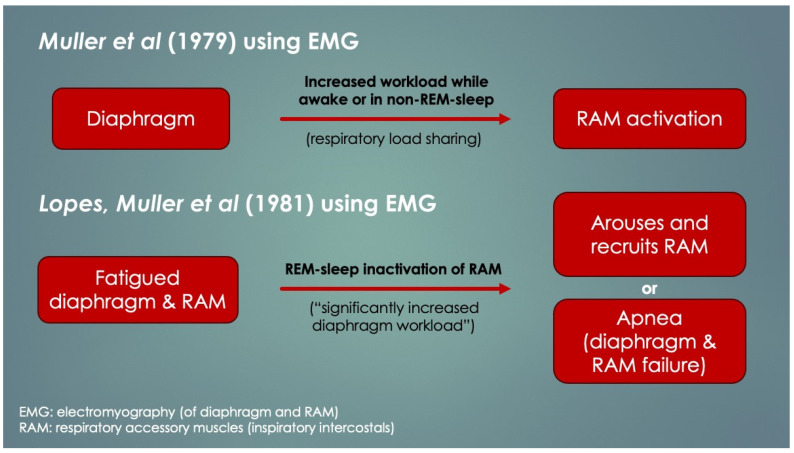
Respiratory load compensation, REM sleep inhibition and arousal versus apnea in infants with diaphragm fatigue. Normally, under increased physiological demand or diaphragm fatigue, ventilatory workload is shared between the diaphragm and RAM (the latter include the inspiratory ICM). However, sudden RAM inactivation in REM sleep leads to apneas related to the already present and consequently worsening diaphragmatic fatigue (causing peripheral failure). Lack of arousal, with no consequent RAM recruitment, potentiates the apneas [4,5,6].

Another crucial paper was by Lopes et al. (1981) [4], who determined diaphragm fatigue existed in 12 of 15 otherwise healthy preterm infants at mean 19.9 ± 13.7 days age using surface electromyography (EMG) of the diaphragm and intercostal muscles (ICM). Moreover, two different patterns of response to fatigue were observed: as opposed to infants who recruited their ICM, apneas occurred in those that did not. Some infants with prolonged apneas required tactile stimulation (presumably to wake them to breathe), and failing that, short-term CPAP and even mechanical ventilation (MV). Normally, respiratory accessory muscle (RAM) recruitment, which includes the ICM, occurs by a process known as respiratory load sharing (or load dependence), wherein ventilatory workload is diverted from the diaphragm to ICM as diaphragm fatigue sets in (and vice versa) [5]. In fact, rib retractions, commonly observed in children with respiratory distress, provide a direct visual cue of ICM activation secondary to diaphragm fatigue. Another physical sign of diaphragm fatigue (and outright failure) is paroxysmal breathing or thoraco-abdominal asynchrony. It also occurs in diaphragm paralysis and paresis, whereby ventilation is powered or assisted by RAM contractions, respectively. With inspiration, the abdomen becomes drawn in instead of descending and expanding. EMG had determined the apneas involved simultaneous failure of both ICM and diaphragm (preceded by worsening fatigue), and it occurred only when ICM did not take over the work of breathing. The authors discussed the role of REM sleep—when there is physiologic CNS inhibition of skeletal muscles including the ICM but not diaphragm—however, it could not be concluded because sleep state was not measured. Nevertheless, in extension of this line of interpretation, it appears REM sleep predisposed to the fatigue-associated apneas, and they had occurred by failure of both ICM and diaphragm (that is, *peripheral* malfunction). Furthermore, those who had arousal, presumably by hypoxia- or hypercapnia-mediated chemoreception (or tactile stimulation), probably reactivated their ICM, thereby avoiding the apnea. What remains undetermined however, was the reason for the lack of arousals in others as well as the mechanism of diaphragm failure itself. Lastly, had traditional sleep study chest impedance belts been used, these apneas would have been classified as central, because of absent respiratory movements. Figure 2 summarizes these findings and is consistent with other authors’ conclusions [6,7,8].

In support of peripheral apnea in SIDS was evidence presented by Siren and Siren (2011) in their SIDS-Critical Diaphragmatic Failure hypothesis (a review article). They postulated that several diaphragm fatiguing (and added workload) factors cumulatively increase the risk of diaphragm failure in SIDS, including non-lethal viral infections, prone positioning, male sex, hypoxia, hypercapnia, high altitude, bottle feedings as well as underdeveloped RAM and their inactivation in REM sleep [8,9,10,11]. Others included hypomagnesemia, overheating and tobacco smoke. Importantly, all are SIDS risk factors. In support of DCC here though is the sudden and unpredictable nature of SIDS. Suddenness, without prodromal respiratory distress, is in support of a DCC event rather than gradual respiratory failure. Regardless, the Lopes group put it best in 1981 as, “The neural [CNS] basis for apnea is so deeply entrenched that it is difficult to accept that some apnea may be due to [peripheral] respiratory muscle failure”.

### 2.3. Diaphragm Hyperexcitability Disorders (DHD)

Further literature review revealed numerous contraction abnormalities of the diaphragm, most importantly, diaphragm flutter (van Leeuwenhoek’s disease) [12] and respiratory flutter. Diaphragm flutter (DF), thought to be rare, involves rhythmic involuntary contractions of the diaphragm superfluous to those that occur with normal neural (CNS-mediated) autonomic breathing (as opposed to volitional breathing, which involves a separate neuroanatomic pathway also carried by the phrenic nerves). Respiratory flutter denotes excitation of the accessory muscles such as the rectus abdominae and ICM in addition to the diaphragm. Immediately, its existence alone demonstrated how anatomically disparate groups of inspiratory muscles can simultaneously develop pathological excitation (supporting the hypothesis that cramping of both diaphragm and ICM caused the case patient’s symptoms) [13]. In that paper, respiratory flutter in three full-term neonates was reported as an “underrecognized cause of respiratory failure”, diagnosed by respiratory inductive plethysmography (RIP) and fluoroscopy. Within hours of birth, all developed ventilatory distress involving stridor, grunting, rib retractions, and inspiratory ratchetlike or fluttering chest movements requiring temporary CPAP or MV. Chlorpromazine, a typical antipsychotic medication, helped abate the flutter in all three. However, in another report, DF was well tolerated in three babies of mean 12-weeks’ age (gestational age unknown) with bronchopulmonary dysplasia and respiratory syncytial virus (RSV) bronchiolitis [14]. Notably, all are SIDS risk factors (including young age), suggesting a possible link between flutter and SIDS. Aside from postconceptional age though, it is uncertain why there was such a difference in clinical presentations between the patients reported in the two papers.

**Figure 3:**
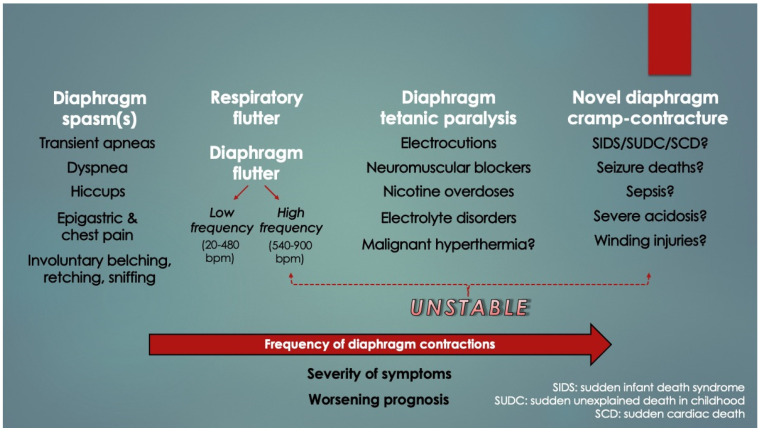
Spectrum of diaphragm hyperexcitation disorders (DHDs). Polyonymous clinical descriptors and diagnoses upon literature review made it a challenge categorizing them. However, a pattern emerged: as the frequency of non-physiologic diaphragm contractions increased, respiratory distress became prominent, some with sustained apneas (respiratory arrest). Those to the right presented with severe respiratory distress or frank arrest and could be termed, “unstable diaphragmatic arrhythmias”. DCC could belong to this spectrum.

Numerous case reports of other pathological excitation states (neuromuscular irritability) of the diaphragm were found on review, and these were broadly classified under single contraction phenotypes (e.g., hiccups and spasms) [15,16] or arrhythmias (e.g., low and high frequency flutter and fibrillation) [17,18]. Examples of such hereby termed “diaphragm hyperexcitable disorders” (DHD) in addition to hiccups, spasms, flutter, and fibrillation were diaphragmatic and respiratory tics, fasciculations, palpitations, myoclonus, tremor and Belly Dancer’s dyskinesia. Summed up, however, it can be seen in Figure 3 that DHDs appear to present along a frequency spectrum of worsening symptoms and prognoses. The higher the frequency, the more clinically unstable is the patient. This is a novel finding. DCC, which could represent a very high-frequency DHD akin to tetany, might also belong to the spectrum; however, given extremely high mortality with few survivors, these cases would have inadvertently been excluded by clinical studies (an inverse form of survivorship bias, or mortality bias).

Tobacco smoke exposure is a well-known SIDS risk factor. Nicotine poisonings in young children, even from ingesting snuff box scrapings, have led to rapid deaths from respiratory arrest (under 30 min). It was not centrally mediated [19]. Rather, it occurred by peripheral paralysis from a sustained diaphragmatic spasm (essentially a tetanic cramp-contracture) [20,21]. Therefore, in infants exposed to subtoxic nicotine levels from household tobacco smoke, it is possible the threshold for diaphragm excitation is lowered (by respiratory muscle fatigue) [22]. Similarly, nondepolarizing neuromuscular blockers like succinylcholine, commonly used to facilitate endotracheal intubation, also induce peripheral respiratory arrest; therefore, both were placed at the most severe position in the spectrum (under tetanic paralysis). In addition, some fatal electrocutions also occur by tetanic contractions of the respiratory muscles (not just cardiac arrhythmias); thus, this too was included [23,24]. Deaths from severe metabolic acidosis (in diabetic ketoacidosis [25,26] and lactic acidosis [27]), malignant hyperthermia [28], botulism [29], rabies [30] and tetanus [31] also appear to occur by peripheral respiratory arrest, even sepsis too (vide infra). Notably, the heart also demonstrates sensitivity to acidosis, as demonstrated by a decrease in threshold for ventricular fibrillation [32].

The etiologies of DHD are extensive and beyond the scope of this paper; however, it is important to note that pain and psychological distress are inciting causes at all ages [17]. Feeding young children can be difficult at times, sometimes requiring forced effort, and this strenuous activity has been associated with apneic, SIDS-like awake deaths [33]. Similarly, hypoxemic episodes in infants occurred with feeding as well as with anger, handling and noxious triggers like pain, airway suctioning, and loud noises [34,35]. This is in striking similarity to life-threatening cyanotic episodes reported in six infants with histories of recent seizures (suggesting a common mechanism with SUDEP; vide infra) [36]. All of this supports the notion that stress-induced diaphragm excitation could cause serious respiratory symptoms, sometimes fatal. [Interestingly, by extension, increased diaphragmatic muscle tone in high-stress situations could physically squeeze on the traversing esophagus (and stomach), leading to the commonly experienced gastric symptoms of anxiety, including “butterflies”, epigastric discomfort, nausea, vomiting, belching and dyspepsia. This would represent a novel connection between mind and body].

Table 1 lists the various causes of respiratory muscle (Type II, hypercapnic) failure obtained by literature review.

**Figure 4:**
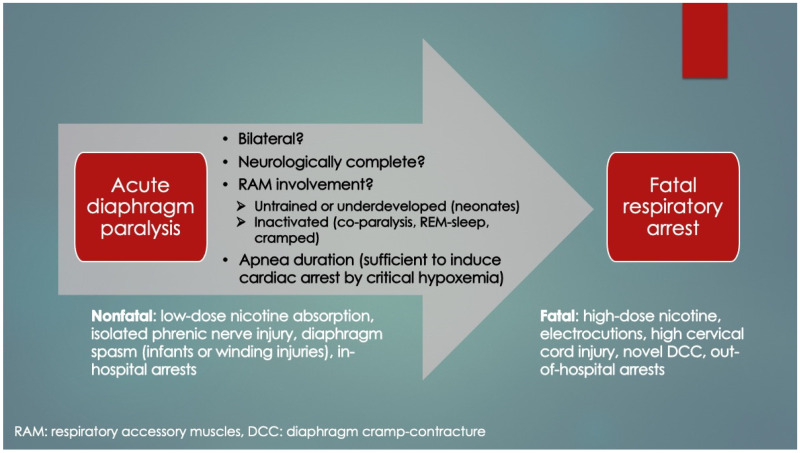
Criteria for fatal respiratory arrests by acute diaphragm paralysis, with examples.

Importantly, it was recognized that diaphragm-related respiratory insufficiency and failure was an underrecognized cause of serious morbidity and mortality in all ages, ranging from fatal soft tissue injuries of the neck (acute bilateral phrenic neuropathies) [53] to heat stroke/hyperthermia [28,73], and terminal COVID-19 infections (acute diaphragm myopathy) [47]. Other important causes included septic, hypovolemic, and cardiogenic shock (vide infra). Categorization could be made by organ involvement (CNS, phrenic nerve(s), diaphragm, or accessory muscles) as well as laterality, rapidity of onset and degree of weakness (complete paralysis versus paresis). For example, whereas some electrical injuries and cervical spine transections induced simultaneous, sudden and complete bilateral paralysis of both diaphragm and RAM, isolated diaphragm weakness occurred by direct abdominal trauma or phrenic nerve injuries (e.g., nerve tractions in birth trauma or chiropractic manipulations). In the very young (neonates), acute bilateral diaphragm paralysis (DP) despite functional RAM, induced critical Type II failure that was followed within minutes by cardiac arrest and death unless reversed by ventilatory support [54,59,60]. This supports the argument that the very young, functional RAM cannot independently ventilate the lungs (when the diaphragm has failed). However, older infants survived probably because their maturing RAM tolerated the added workload. This also provides a compelling explanation why infants aged 2–4 months are at highest risk for SIDS (and not older): underdeveloped, weaker RAM. Older infants have had time for RAM maturation through load-dependent recruitment and training. With this classification scheme, it became evident that acute DP can be immediately fatal when it is bilateral, neurologically complete and occurs in those with weak, paralyzed or cramped RAM (Figure 4). Also, apnea duration needs to be sufficient as to induce critical hypoxemia and secondary cardiac arrest (only 1–2 min) [74]. These too are novel findings.

Death by DCC in young infants satisfies these criteria because the RAM are weak and the effective DP is bilateral and probably complete (i.e., diaphragm fully inactivated by contracture). It is also paroxysmal (sudden and unexpected), just like SIDS and many other child deaths. Lastly, the process is silent, rapid and unwitnessed in most cases (obviating resuscitation efforts).

Table 2 similarly lists the causes of diaphragm fatigue (and increased ventilatory workloads) in infants ascertained upon literature review. Importantly, most, if not all, were known SIDS risk factors (or closely related to them). It should also be pointed out that, as opposed to diaphragm weakness, diaphragm fatigue is reversed by rest. Diaphragm fatigue or dysfunction (DD) is also referred to here as diaphragm insufficiency.

### 2.4. Diaphragm Excitation in Infants with Respiratory Instability

The greatest pathologic threat to infants is respiratory, and sleep is an especially vulnerable state. This is particularly concerning not only because they disproportionally spend their time sleeping, but most of it is active (REM) sleep, a stage associated with more respiratory instability. In living infants, this is observed as transient apneas and hypopneas (shallow breaths) as well as periodic breathing, increased arousals and hypoxemic episodes (HE). 

Recurrent HE predispose to serious long-term morbidity such as cerebral palsy, retinopathy, blindness, deafness, poor growth, neuro-developmental delays and pulmonary hypertension with cardiomyopathies. Mortality is higher, including by sudden respiratory arrests in RSV bronchiolitis [91] and sudden unexpected deaths, even as late as 18 months of age, if not longer (consistent with SIDS and SUDC, respectively) [35,120]. It is also more common in those with chronic conditions such as bronchopulmonary dysplasia, cerebral palsy and neuromuscular diseases, like muscular dystrophies and congenital myopathies.

Some HE are not visually apparent, whereas others may exhibit cyanosis, pallor, hypotonia, loss of consciousness or seizures. Simultaneous behaviors include *silent* squirming, kicking, grimacing, writhing and bearing down, sometimes followed by hiccups (diaphragmatic contractions) [34]. Unusually, these behaviors are associated with crying, so the silence supports the case patient’s report that inspiratory arrest had occurred. This is corroborated by the work of Southall et al. (1990) [35], who found prolonged absence of inspiratory efforts was common among 51 infants with recurrent cyanotic HE. Expirations still occurred, also consistent with our case. It is important to point out that that most HE are not preceded by seizures.

Defined as oxygen saturations under 88% for over 10 s, HE in one study lasted from tens of seconds to two minutes, occurring up to hundreds of times daily in spontaneously breathing, former preterms at 44 ± 21 days of age [121]. They even occur in infants receiving mechanical ventilation, as exhibited by forced expirations, hypopneas and reliance on ventilator breaths associated with doubling of pulmonary resistance and reduced lung compliance of unknown origin (with simultaneous increases in gastric and esophageal pressures) [122].

**Figure 5:**
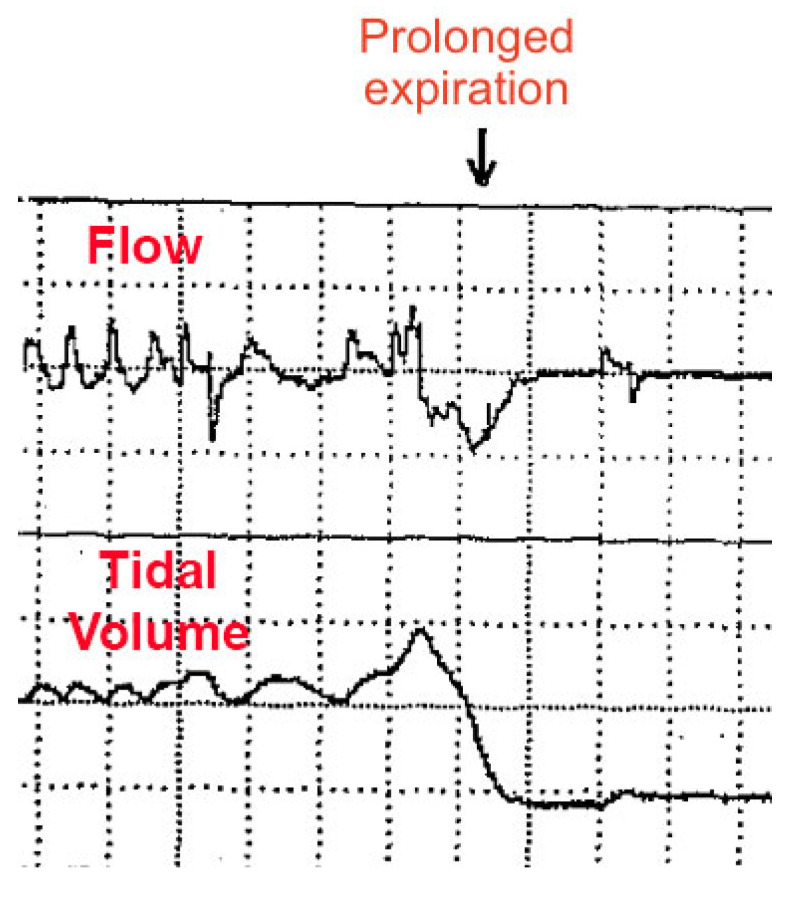
Prolonged expiratory apnea in an infant. The apnea (seen as flatlines in both flow and tidal volume waveforms) was preceded by a big expiration with a decrease in lung volume. Typically, this is followed by a hypoxemic episode (transient oxygen desaturations).

Respiratory instability is thought to be caused by abnormal chemoreception and immature development of the neural control of breathing, worsened by underdevelopment of the lungs (primarily, surfactant deficiency). However, some HE appear to occur by “active” (voluntary) abdominal muscle contractions (causing forced expirations and hypopneas), sometimes followed by breath-holding (short apneas) [123,124]. As seen in Figure 5, an abnormal respiratory waveform can manifest: a prolonged expiration (or prolonged expiratory apnea). Importantly, this occurs just at the end-expiration phase of the respiratory cycle (identical timing to DCC onset as reported by our patient). 

Curiously, almost nothing is mentioned in the literature about the vital muscles that power respiration, despite compelling reports from the late 70’s indicating infants were “very close to the threshold of fatigue” [and failure] [6]. Diaphragm fatigue occurs by a number of factors unique to very young compared to older infants: (1) highly compliant, cartilaginous ribcage, which reduces ventilatory efficiency; (2) less efficient RAM contributions because of less conditioning and anatomical differences (worsened in REM sleep by CNS inhibition); (3) decreased range of pump displacement due to diaphragm flattening; (4) fewer fatigue-resistant, slow-twitch diaphragm myofibers; and (5) lower total cross-sectional area of all myofiber types [75]. Also, at any age, anything that increases work of breathing (or respiratory rate), like REM sleep, a roll to prone position, pain, psychological distress, forced bottle feedings, laryngospasm, bronchospasm, or pulmonary infections, exacerbates the fatigue, even sepsis and severe acidosis (evident as Kussmaul’s hyperpneic, labored respirations to “blow off” CO_2_). The latter two also directly impair diaphragm contractility and relaxation (vide infra). In addition, anemia, a known risk factor for apnea of prematurity [125], as well as apparent life-threatening events in infants [112] and breath-holding spells in older children [126], could explain these apneas simply because the reduced blood oxygen-carrying capacity exacerbates DD, particularly under times of increased physiologic demand. Clearly, the causes of DD-induced respiratory instability in infants (and proposed ventilatory failure) are complex and multifactorial, in keeping with the host of events thought to lead up to a sudden unexpected infant death.

Another held mechanism of HE is hypoxic (and hypercapnic) ventilatory depression (HHVD). The process, exacerbated in REM sleep, is thought to be from central causes. Normally, the compensatory (“CNS feedback”) response to hypoxemia or hypercapnia in mammals is by an increase in minute ventilation (V_E_ = respiratory rate x tidal volume). This happens in both adults and infants, but after an initial 1–2 min compensatory increase in infants (and about 20 min in healthy adults, depending on degree of hypoxia or hypercapnia), it is suddenly followed by a prolonged decrease (or depression) of V_E_ by a reduced respiratory rate. It has been a mystery why this presumed neural response is dysfunctional. Instead, it hereby appears HHVD might actually be caused by the reduced work output of diaphragm fatigue (insufficiency). It just takes longer to appear in adults because their mature, conditioned respiratory muscles are less vulnerable to fatigue. The process is simple: persistent hypoxia or hypercapnia (e.g., from breathing exhaled gases or hypoventilation) combined with acidosis (from both respiratory and metabolic types) induce DD, leading to reduced alveolar ventilation, which further compounds the hypoxia, hypercapnia, and respiratory acidosis in positive feedback cycles. The slow onset yet progressively worsening hypoxia of an unstable DD feedback cycle during sleep would explain the hypoxemic gasping seen in the Poets’ SIDS victims (where it preceded gasping, bradycardia and apnea alarms) [3]. As we shall see, repeated HE by this mechanism could be responsible for myopathic changes seen in the diaphragms of SIDS victims.

**Figure 6:**
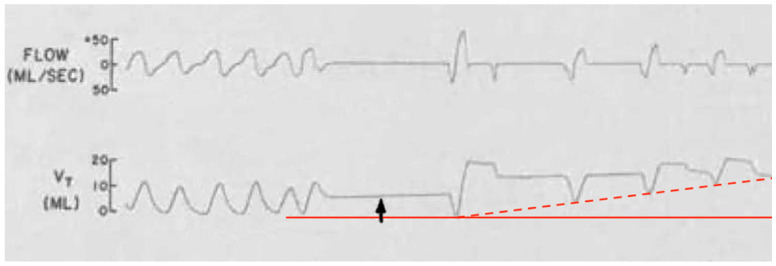
Partial Sleep polysomnograph demonstrating air trapping (breath stacking), hyperinflation and slowed, reversed respirations after an unidentified apneic event in a spontaneously breathing infant (the full image can be seen in Figure S1). Normal tidal breathing was interrupted by a brief apnea (arrow) associated with silent squirming. It was followed by a stepwise, breath-to-breath increase in end-expiratory volume consistent with breath stacking (compare broken red line to solid red, tidal breathing baseline). Unusually, these respirations were slowed and reversed, in which expiration preceded inspiration (seen in the airflow tracing). All such changes were associated with persistently increased esophageal pressure for 44 s (not shown), thought secondary to voluntary abdominal muscle contractions (measured by surface EMG). Instead, diaphragm spasm could have been responsible, cross contaminating the EMG. Reprinted with permission of the American Thoracic Society. Copyright © 2024 American Thoracic Society. All rights reserved. The American Journal of Respiratory and Critical Care Medicine (previously known as The American Review of Respiratory Disease) is an official journal of the American Thoracic Society. From: Abu-Osba YK, et al. (1982) [34].

In vivo analyses of ventilatory mechanics have determined that the dysfunctional drop of respiratory rate in HHVD occurs by a prolongation of the expiratory phase (T_E_) of the breathing cycle. This too is thought secondary to CNS immaturity. However, in hamster diaphragm strips ex vivo, Esau (1989) [107] determined both hypoxia and hypercapnia reduced diaphragm contractility and slowed the muscle relaxation phase (T_R_) of diaphragm contraction. By extension, this could be responsible for the delayed T_E_. Similarly, Herve et al. (1988) [127] found in rat diaphragm strips that increased workloads as well as ryanodine, an inducer of muscle fatigue, both delayed T_R_. And when overloaded by ryanodine, contractility was markedly lowered, and a diaphragm *contracture* developed (excitation). Nicotine exerts a similar effect in toxic doses [20,21]. Although not reproduced in vivo, intact diaphragms might also be prone to contracture when T_R_ becomes longer than T_E_ (causing spasms and cramps). This could occur in a fatigued diaphragm working under higher respiratory rates and ventilatory workloads (both common in illness), when it is unable to fully relax and return to its original resting position in time for the next breath. Literature evidence supporting this notion was limited; however, Figure 6 demonstrates an example. There is an upwards wander in the chest impedance (tidal volume, V_T_) baseline with air trapping (hyperinflation). It occurred in a spontaneously breathing preterm infant immediately after a short apnea associated with silent squirming and a spike in surface EMG activity thought to be from abdominal contractions. Instead, diaphragm spasm could have been responsible. By comparison, air trapping in spontaneously breathing individuals does happen in COPD, asthma, and interstitial lung diseases; however, does not apply here given the patient’s age.

Along with hypoxia, hypercapnia, ryanodine, fatigue and possibly nicotine, both acidosis and endotoxins of *S. Pneumoniae* and *E. Coli* have been found to also prolong diaphragmatic T_R_, precipitating diaphragm contracture ex vivo [107] and further fatigue in vivo [95]. This could be important not only in causing respiratory instability in infants with bacterial infections, but also sudden respiratory arrests in SIDS, SUDC and all other age groups. Both respiratory acidosis and metabolic acidosis (the latter for example from hypovolemia-shock as well as upper respiratory infections and diarrhea-associated bicarbonate loss) [128] overlap with SIDS risk factors, including intercurrent infections, dehydration, fever and overheating. They could manifest in deadly fashion at the diaphragm by sudden respiratory failure from contracture. In fact, extreme acidosis and hyperkalemia at SIDS autopsies were reported in a 2006 online Medscape article but unfortunately were never confirmed [43]. Electrolyte disorders can affect the diaphragm much in the same way as the examples above, by inducing fatigue, but in this case from excitation-contraction coupling dyshomeostasis. The author went on to mention how critical acidosis could have developed in the days preceding the deaths. Both disorders were also identified in a report of 20 infants with idiopathic postneonatal apnea occurring in association with hypomagnesemia [129]. The sickest ones demonstrated bradycardia, acute respiratory distress and skeletal muscle hyperirritability; however, the diaphragm itself was not investigated. That author later went on to suggest hypomagnesemia causation in SIDS. Severe hypokalemia, too, is a known cause of respiratory muscle paralysis leading to asphyxiation [44]. Finally, parenteral nutrition, commonly administered to undernourished neonates, carries a risk for metabolic acidosis [130] and thus potential for fatigue-excitation. In summary, it appears hypoxia, hypercapnia, acute acidosis, endotoxins, nicotine and electrolyte disorders all intersect at the diaphragm in infants, altering breathing mechanics (reduced contractility and delayed relaxation), culminating in fatigue and excitation in the form of spasms and cramps with consequent apneas and other forms of respiratory instability, leading to HE.

Like the limbs, the diaphragm is composed of skeletal muscle. Fatigued skeletal muscles develop increased tonicity and neuromuscular excitation under increased workloads, commonly experienced when one is unfit, dehydrated or overheated [84]. Examples of muscle excitation in vivo include twitches and fasciculations, spasms and cramps (sustained spasms), myoclonus (short arrhythmic spasms), and arrhythmias like flutter and fibrillation (which are well known to affect the heart). Pathological excitation of respiratory muscles has already been described, as evidenced by diaphragmatic and respiratory flutter. Importantly, whereas apnea from diaphragm spasm is not life-threatening because of its transience, that from a sustained cramp could be. With persistent diaphragm cramp-contracture in an infant, severe hypoxemia would ensue, causing sudden hypotonia, cyanosis and possibly seizure, followed by bradycardia and cardiac arrest and death if not rapidly aborted. Unlike the 7-year-old case patient who autoresuscitated by troubleshooting and learning to breathe *in reverse*, infants are clearly not capable of such a complex, counterintuitive task, thus a cardiopulmonary emergency could arise. Finally, whereas limb muscle cramps can be aborted by active or passive stretching, this is not clearly possible with the internally located diaphragm. However, it is interesting to consider how the patient’s rescue breaths might have terminated his DCC. Perhaps the rapid combination of expiration and inspiration forced the diaphragm to initially recoil cranially and then quickly stretch as it moved caudally with inspiration, thereby overcoming the contracture tension and returning to normal function.

In infants with respiratory instability from diaphragm fatigue, diaphragmatic spasms with consequently delayed relaxation (akin to prolonged cardiac repolarization in diastole after premature ventricular contractions occur) are hereby proposed to cause the observed forced expirations and breath-holding pauses, respectively, along with hypopneas and prolonged apneas. Diaphragm spasms would mimic abdominal muscle contractions seen on surface EMG. They too could increase esophageal and gastric pressures (diaphragm moves cranially, thereby reducing thoracic volume). This is supported by the diaphragm/RAM-induced apneas of the Lopes study [4] and provides an alternative explanation of HE, ridding any notion of a voluntary or behavioral component to forced expirations and breath-holding. It is also more intuitive given it is unlikely an undernourished, feeble preterm infant would be capable of persistently tensing their abdomen in one instance for over two minutes, to the point of dropping their oxygen saturations below 75% [121]. Diaphragm spasms could also explain the silent nature of HE simply because an inspiration cannot be taken while the organ is inactivated. Furthermore, the pain of spasm would explain the grimacing, writhing, and kicking. Finally, this provides a solid foundation to hypothesize that persistent spasm, or diaphragm cramp-contracture, could be responsible for non-arrhythmogenic sudden unexpected deaths in infants (and possibly other ages too).

**Figure 7:**
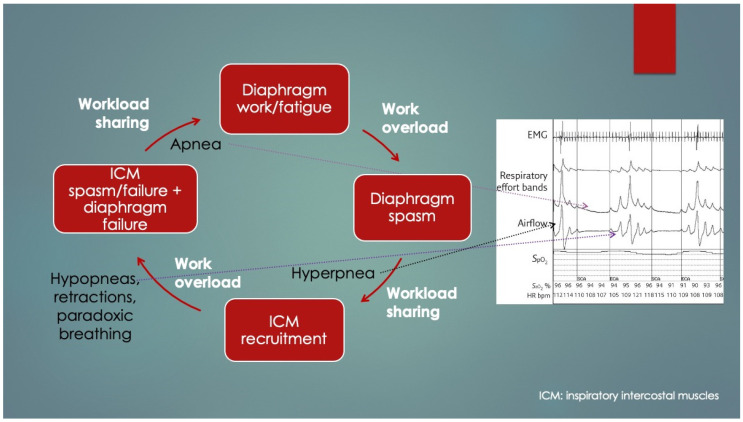
Periodic breathing is hereby proposed to involve cyclical diaphragm fatigue and spasms alternating with intercostal muscle fatigue and spasms. Due to respiratory load compensation, ventilatory workload shifts from diaphragm to ICM and back as each muscle group fatigues, fails by spasm, and then recovers. Cyclical spikes in D-EMG, thought here to be diaphragm spasms, occur in association with hyperpneic breaths followed by hypopneas and transient apneas. Hypopneas are thought to occur from independent ICM action, whereas apneas are caused by simultaneous failure of both diaphragm and ICM. *Insert*: Periodic breathing in an infant. Courtesy of Dr. Sadasivam Suresh, Dept. of Paediatric Respiratory and Sleep Medicine, Queensland Children’s Hospital, Brisbane, Australia.

It follows then that periodic breathing also involves fatigue of the diaphragm that becomes temporarily dependent on the accessory muscles to maintain ventilation until reversed by rest (and vice versa by load sharing). Specifically, cyclical episodes of diaphragm fatigue, work overload and consequent spasms with transient inactivation followed by load compensation by RAM activation and then RAM fatigue and spasms would give rise to a repetitive sequence of hyperpneas (from diaphragm spasm), hypopneas with paradoxical breathing (by independent RAM contractions while the diaphragm is temporarily inactivated) and apneas (simultaneous failure of both diaphragm and RAM) commonly observed and as seen in Figure 7 [131]. Seppä-Moilanen (2019) [132] determined periodic breathing was substantially reduced with supplemental oxygen and caffeine in 21 preterm infants. Apneas were also reduced in frequency. Both interventions may have improved diaphragm function directly rather than by centrally mediated effects. Aubier (1989) flatly summed this up, stating, “It is clear that the majority of chest physicians have emphasized disorders of the lung or abnormalities of ventilatory control while ignoring the muscles”.

### 2.5. Diaphragm Fatigue, Excitation and Obstructive Sleep Apnea

**Figure 8:**
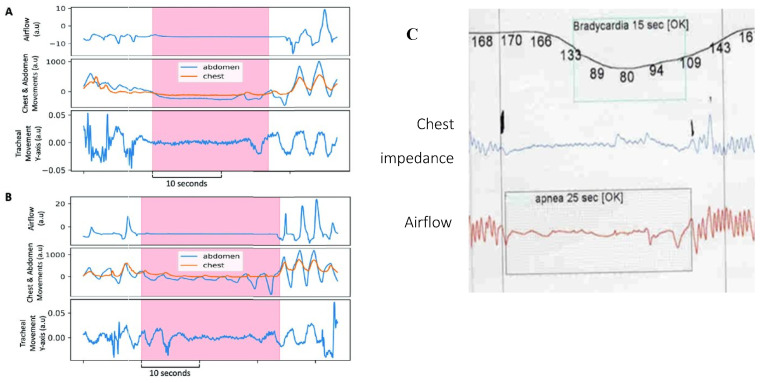
(**A**–**C**). Central versus obstructive sleep apneas measured by airflow and impedance plethysmography. Central and obstructive apneas (pink shading) may in fact have peripheral (diaphragmatic) origins. (**A**) Central apneas exhibit no airflow and lack of respiratory movements of chest and abdomen. They are presumed to occur by lack of neural stimulation to the ventilatory muscles but could rather be from combined peripheral failure of diaphragm and RAM. (**B**) Obstructive apneas involve no airflow associated with respiratory movements, here seen as reduced-amplitude hypopneas (middle frame). Supra-glottic muscle collapse is thought responsible but could rather be isolated RAM activity breathing against an immobile diaphragm. (**C**) “Central” apnea with flutter-like or fibrillatory chest movements (blue line) with brady-hypopneas (red). Although cardiac artifacts could be responsible, so could respiratory flutter.

Obstructive sleep apnea (OSA) in children and adults is a highly prevalent, frequently underdiagnosed condition. Sleep disorders are often missed by parents or the individual. OSA too carries significant short and long-term morbidity and mortality. Complications include systemic and pulmonary hypertension, cardiomyopathies, congestive heart failure, coronary artery disease, cardiac arrhythmias, stroke, venous thromboembolism, and increased risk for SCD [133]. It is also associated with gastroesophageal reflux, which improves significantly with CPAP; however, the mechanism linking the two has remained unknown [134]. Like HE above, but in older children and adults with OSA, transient diaphragm spasms and compensatory RAM action are hereby proposed to cause the observed apneas and hypopneas, respectively, as seen in Figure 8.

This is not to dismiss the existence of upper airway (supraglottic) obstructions from atonic muscles or enlarged tonsils. In fact, they contribute to DD because of the increased work of breathing from added airway resistance. Furthermore, this novel diaphragmatic paradigm of OSA states that obstruction develops when RAM independently attempt to breathe against the immobilized diaphragm inactivated by spasm. This would explain the doubling of pulmonary resistance mentioned above, which was associated with HE in mechanically ventilated infants [122]. It is also supported by Southall’s anecdotal findings of initial resistance to inflating the lungs when resuscitating those with severe HE, even with functioning tracheostomy or endotracheal tubes in situ (maintaining airway patency) [35]. Additional support comes from Miller et al. (1993) [135], who revealed in the breaths immediately preceding and following apneas in preterm infants, there was a stepwise increase in total pulmonary airway resistance not caused by supraglottic muscle collapse. Other evidence linking diaphragm involvement in OSA comes from an EMG study comparing activation of the respiratory muscles, including those of the upper airway, ICM and diaphragm, in adults with OSA with healthy controls [87]. All such respiratory muscles in the test subjects were more active than controls, both awake and asleep, reflecting an added workload. Moreover, with the onset of airway obstruction, there was a breath-to-breath, rapid drop in D-EMG followed by a gradual, then sudden increase with resumption of airflow a few seconds later. This was mirrored by similar changes in transdiaphragmatic pressures. The reduction was thought to be from reduced neural drive and an inhibitory reflex but could rather have been from diaphragm fatigue and spasm. It appears once the diaphragm had recovered from spasm it was able to resume functioning; however, a higher level of work was needed initially, probably to overcome airway resistance by diaphragm hypercontraction and immobility as well as the resistive elastic forces of pulmonary compliance.

It follows then from chest impedance studies in OSA, that when RAM function independently of a diaphragm inactivated by spasm, low-amplitude hypopnea waveforms are produced. These would mimic obstructive apneas (Figure 8B) and, at bedside, might appear with rib retractions and paradoxic breathing (thoraco-abdominal asynchrony). By contrast, when both diaphragm and RAM are simultaneously inactivated (e.g., by diaphragm spasm from REM sleep RAM inactivation, or “co-spasms” akin to respiratory flutter), near-flatline apneas appear, mimicking central apneas (Figure 8A,C). Once spasms finally resolve, ventilations would resume (but initially at higher intensity). Indeed, upon careful scrutiny of impedance, flow and D-EMG waveforms in central apneas, most often seen is a fine, tremulous baseline. This could represent highly attenuated diaphragm electrical activity by pathological excitation. In fact, many central apneas in one D-EMG study were determined to be from another cause [88].

**Figure 9:**
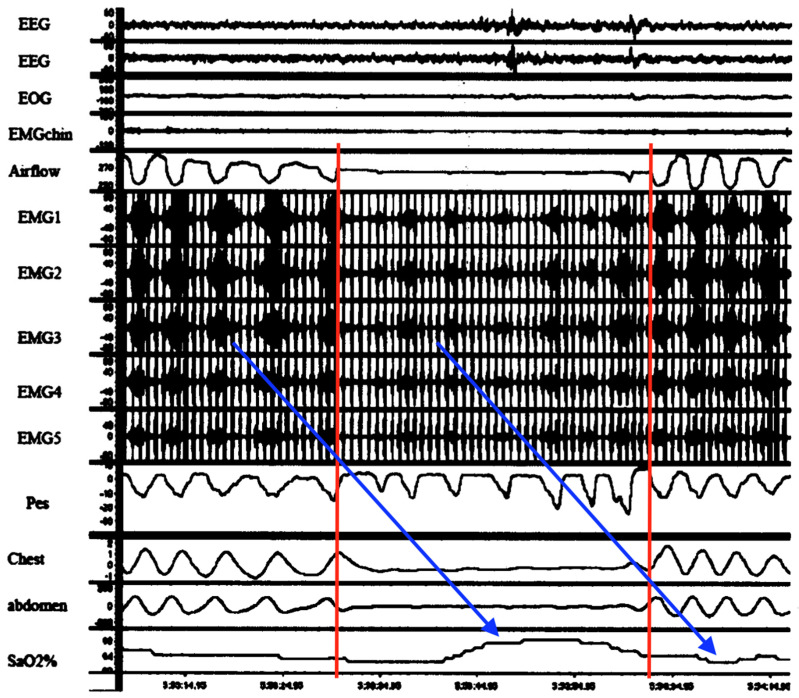
Evidence for diaphragm electromechanical dissociation in a sleep apnea (esophageal D-EMG1-5). Blue arrows show the delay in oxygen desaturations after a pathological event involving the diaphragm that exhibited periodicity. The apneic period occurs between the two red lines where there was no airflow or respiratory movements (seen in RIP chest and abdomen). This would therefore be classified as a central apnea. However, there was D-EMG neural activity (albeit attenuated); thus, it is not central. Obstructive sleep apnea would then be presumed; however, that normally involves continued breathing movements, of which there were none. Instead, something else was impairing ventilations associated with reduced D-EMG (i.e., diaphragm spasm). Continued esophageal pressure (P_es_) swings during the apnea could have been caused by independent action of the accessory muscles, while the attenuated D-EMG from continued neural activity during the spasm. It appears a complete novel airway obstruction developed by RAM attempting to inspire against the temporarily inactivated, immobilized diaphragm. Airflow resumed, but with increased D-EMG activity, presumably to overcome airway resistance. Reproduced with permission. Source: Luo et al. [88].

Figure 9 is very important because it temporally connects abnormal diaphragmatic electrical activity on intra-esophageal D-EMG in an adult apnea with a complete lack of ventilatory movements. It was neither a classic central, obstructive nor even mixed apnea. Although cause and effect cannot be ascertained, there was significant attenuation of D-EMG amplitude (and frequency) during the episode. Combined with the lack of respiratory movements, this is consistent with diaphragm inactivation due to spasm (electromechanical dissociation). Respirations resumed but only with higher intensity electrical bursts (perhaps from increased neural drive and higher diaphragm work to overcome post-obstruction airway resistance). Also, although truncated, D-EMG and oxygen saturations exhibited periodicity, suggestive of respiratory load cycling with RAM. Lastly, this evidence suggests that D-EMG activity reflects not just neural drive, but rather a composite influenced by diaphragm electromechanical output.

**Figure 10:**
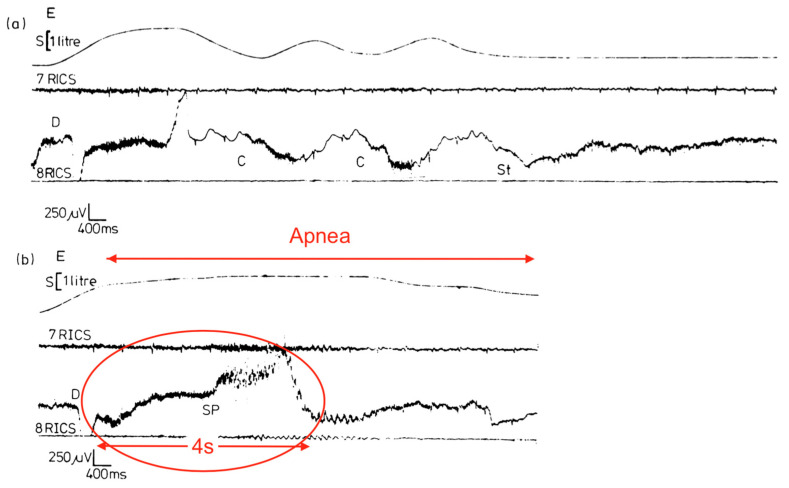
Spirometry (top, “S”), intercostal EMG (“7,8 RICS”) and diaphragm EMG (“D”) during a generalized spasm in a tetraplegic adult with dyspnea. Continuous tracing from (**a**) to (**b**). Breathing stopped in association with coughing (C), straining (St) and a diaphragmatic spasm (SP; red ellipse). This would otherwise have been deemed a central apnea. It persisted longer than the spasm, presumably due to delayed recovery of diaphragm function. 7RICS: seventh right intercostal space. With permission by BMJ Publishing Group Ltd. license (August 2024) [51].

A final (and rare) piece of evidence linking diaphragm hyperexcitation with abnormal breathing was found in a case report of three tetraplegic patients with generalized body spasms triggered by deep breaths or sudden body movements (both increase ventilatory workload) [51]. Using esophageal D-EMG, Figure 10 demonstrates the electrical waveform of a diaphragm spasm with a prolonged apnea. Apnea duration was longer than the spasm, supporting the notion of continued diaphragm inactivity by delayed relaxation (or resetting). Interestingly, symptoms occurred more often in colder environments and if anxiety was present (the former a SIDS risk factor, while the latter a known trigger of DHD and HE).

Nocturnal sweating is another known SIDS risk factor and hypothesized here to be secondary to overheating from increased diaphragm work, exacerbated by fevers and overwrapping in bed. In an Icelandic study of adults with OSA, frequent night sweats (≥3 times/week) were reported by 30.6% of male and 33.3% of female patients, compared with 9.3% of men and 12.4% of women in the general population (*p* < 0.001) [136]. Boys are also more likely to have night sweats than girls [83], potentially reflecting an increased work of breathing. Evidence to support harder working, fatigue-prone respiratory muscles in boys compared to girls includes (1) significantly thinner diaphragms on ultrasound in preterm [97] and adult males [77], (2) a faster rate of diaphragm fatigue along with lower inspiratory endurance times based on transdiaphragmatic pressures in adult males [78], and (3) higher overall respiratory morbidity and mortality in preterm males [79]. Additionally, women demonstrated greater recruitment of ICM compared to men due to anatomic rib differences, thus reducing diaphragmatic workload [80]. Lastly, male neonate mice exposed to in utero asphyxia for 7.5 min had decreased survival at one hour after birth compared to females (survival rates 52% and 69%, respectively) [55]. Their diaphragms demonstrated significantly worse structural and functional deficits (reduced maximum tetanic force and fatigue resistance), persisting long-term in those that survived, but associated with higher morbidity and mortality. All these findings provide a compelling explanation for the male preponderance of SIDS cases.

Another important point about night sweats is the potential perils of polar fleece and other synthetic fabrics commonly used in children’s bedding and clothing. Essentially, these do not breathe (ventilate heat or wick body moisture) like natural fabrics do [137]. Overheating might result, leading to increased risk of SIDS and SUDC, especially if febrile and over bundled. This could reduce the DCC threshold by increasing diaphragm fatigue or workload.

### 2.6. Peripheral Respiratory Failure in Septic and Cardiogenic Shock

Diaphragm muscle fatigue and failure have been reported in septic Mongrel dogs. In ten spontaneously breathing anesthetized animals given intravenous *E. coli* endotoxin, all died within 4.5 h by respiratory arrest. Hussain et al. (1985) [74] determined by transdiaphragmatic pressures and EMG of diaphragm and ICM that all cardiac arrests were preceded by rapidly progressive diaphragm muscle fatigue and sudden failure. This refuted traditional thought, that CNS depression coupled with severe lung disease was the cause of alveolar hypoventilation, severe hypoxemia, and death. Unfortunately, such techniques could not reveal if diaphragm excitation had occurred. However, the authors mentioned how diaphragmatic failure was not unique to sepsis, as it had also occurred in animals with cardiogenic shock induced by cardiac tamponade. That was in reference to Aubier et al. (1981) [138], who had injected saline into the pericardial cavities of 13 spontaneously breathing adult dogs compared to seven on MV. Like severe sepsis, deaths occurred quickly, within 2.5 h, in all those of the former group secondary to progressive diaphragm fatigue and sudden failure. Electromechanical dissociation had occurred whereby neural drive was maintained to the diaphragm (as measured by phrenic nerve root electrodes), but the respiratory muscles failed as force generators. Three possible causes were outlined: (1) blockage of nerve impulses at the neuromuscular junction; (2) impairment of excitation–contraction coupling; and/or (3) failure of the contractile machinery itself. The latter was reasoned most likely, in keeping with putative DCC, and probably had occurred from the effects of diaphragmatic hypoperfusion in shock. This would have led to local hypoxemia and a shift to anerobic metabolism within the diaphragm along with lactic acid accumulation contributing to the contractile dysfunction and organ failure. By extension, under high respiratory rates, when there is less time for diaphragm perfusion to occur, local hypoxia and acidosis would be exacerbated [139]. This fatigue–failure process in hypoperfusion is supported by a study in which diaphragm perfusion pressures were directly increased via phrenic artery catheters after diaphragm fatigue was induced in anesthetized, ventilated dogs [140]. It reversed the DD.

A pattern emerges from the above two circulatory shock experiments (septic and cardiogenic), wherein peripheral respiratory fatigue and failure preceded cardiac arrests and rapid deaths in previously healthy, spontaneously breathing animals. Hypotension with reduced diaphragmatic perfusion appears to be the common pathologic mechanism wherein blood supply did not meet the metabolic demands of the organ. Anemia (already discussed) and hemorrhagic shock [114] would worsen this, as would deficiencies of oxygen and substrates like glucose, fatty acids, and electrolytes such as potassium, calcium, phosphorous, and magnesium, which are important in excitation–contraction coupling. Indeed, acute hypophosphatemia has been linked to respiratory fatigue in hospitalized patients [141] and associated with prolonged MV and delayed discharge from the pediatric ICU [142]. More than one author has suggested this in SIDS causation, where hypomagnesemia too has been suspected in causing severe limb muscle weakness worsened by prone position (asphyxia hypothesized from inability turning head to avoid rebreathing) [41]. However, we now have a stronger mechanism that links all such electrolyte and metabolic abnormalities, and it centers on sudden diaphragmatic failure.

As such, in a young infant sleeping at home with dehydration, acute electrolyte disorders, and acidosis from both metabolic and respiratory causes, as well as perhaps diaphragm viral myositis (vide infra)—all compounding hypoxemia and hypercapnia worsened by diaphragm fatigue—it could be as simple as an acute workload increase by REM sleep or a roll to the prone position that suddenly triggers excitation by DCC. Acute bilateral diaphragmatic paralysis and respiratory failure would ensue, followed by RAM activation (if arousal occurs) and a terminal struggle to breathe against the internal airway obstruction produced by the hypercontracted diaphragm. Within only 1–2 min, critical hypoxemia, bradycardia, cardiac arrest, and death would occur. All the while happening silently and without warning, unwitnessed, and with rapid deterioration ultimately leading to a postmortem SIDS diagnosis. Table 3 describes such a sequence of diaphragm fatigue-DCC-respiratory arrest in a hypothetical infant sleeping upstairs in a smoking household in a cold winter climate with artificial home heating.

### 2.7. Diaphragm and Limb Myopathy, Contraction Band Necrosis and Tardieu Spots

It is proposed that respiratory arrest by DCC is responsible for some SIDS and SUDC cases as well as some SCD in adults. Like the novel obstruction of OSA outlined above, where diaphragm spasm mechanically resists RAM contractions (causing transient apneas-hypopneas), sustained apneas would occur in DCC. If not overcome by autoresuscitation or rescue breaths, asphyxia and death will ensue. Unfortunately, like ventricular fibrillation, pathological pump contractions are temporary and do not persist postmortem, making it impossible to confirm at autopsy. However, Kariks (1989) [143] found indirect evidence in the diaphragms of SIDS victims. Although controls were not provided, contraction band necrosis was present in 82% of diaphragms (D-CBN) along with focal-to-diffuse myofiber ruptures (sarcomere disruptions) and fibrous scars. Acute inflammatory cell infiltrates were not seen (suggesting a hyperacute process). Tissue staining revealed that some sort of diaphragmatic hypercontraction injury (causing irreversible sarcomeric spasm) had occurred terminally and acutely under prolonged anoxia, leading to contracted segments of thick and thin muscle filaments. Fibrous scars in various stages of healing suggested prior non-fatal injuries had occurred by repeated HE in the preceding days to weeks. Silver & Smith (1992) [144] confirmed these myopathic findings, stating D-CBN was common in 125 neonates and infants that had died suddenly, primarily by asphyxia. This included birth asphyxia, drownings, suffocation, severe burns with carbon monoxide poisoning and SIDS. Other modes of death included meningitis, head injuries and acute dehydration from severe gastroenteritis [86]. They remarked, “The morphologic age and, if present, stage of healing in each case suggested that the diaphragmatic lesion commenced at or shortly before death or at the time of the cardiac arrest that led to death”. Despite such compelling results, and a few other reports, research in this promising area stalled. Regardless, the evidence makes it imperative to start including diaphragm histology in all autopsies involving sudden unexpected deaths.

A clue to the origin of the diaphragm myopathy in SIDS comes from a study involving acute loading of rabbit inspiratory muscles well above their fatigue thresholds [145]. In other words, extreme exercise. Along with significant hypercapnia and respiratory acidosis (both suggesting diaphragm insufficiency), post-euthanasia sarcomere disruptions with significantly inflamed and necrotic diaphragm tissues was demonstrated in all test animals. Moreover, this occurred in a load-dependent manner (i.e., higher ventilatory workloads correlated to larger areas of myopathy). There was no mention of D-CBN, however, none of the rabbits had died during testing (i.e., presumably no DCC). Interestingly, only 1% of the diaphragm surface area fraction was abnormal, occurring most often in the costal diaphragm and less so in the crural portion and parasternal intercostals. Injured fibers were more widespread throughout the diaphragm in some animals, whereas localized in others. Similar findings were disclosed in 18 preoperative COPD patients exposed to short inspiratory overloads compared to 11 preoperative controls with normal pulmonary function [146]. Intraoperative diaphragm biopsies revealed sarcomere disruptions in all, which were significantly pronounced in the case patients (higher area fractions and densities). Necrosis and inflammatory cells were not observed, possibly due to shorter duration, less intense resistive loading.

In summary, excessive ventilatory workloads exerted load-dependent myopathic changes in the inspiratory muscles of test animals and humans similar to those identified in SIDS. Although little more was found in the literature on the mechanism of D-CBN—and despite no comparative evidence demonstrating muscle cramps as a cause of sarcomere disruption, inflammation or contraction bands in the limbs—progressive diaphragm fatigue culminating in critical excitation is a legitimate CBN candidate. In other words, DCC could be the hypercontraction injury seen in SIDS (and other sudden unexpected deaths). Simultaneous viral infections appear to contribute to the myopathy.

Similar histopathologic changes were reported in children with incapacitating leg cramps associated with viral influenza, predominantly serotype B. Conducted by retrospective analyses of hospital cases of influenza-associated myositis (IAM) as well as case reports and reviews articles, Agyeman et al. (2004) [147] found that calf muscles alone or together with other limb muscle groups (undisclosed types) were involved in 69% and 31% of a combined 316 cases, respectively. There was a gender ratio of 2:1, male–female, in these school-aged children of median 8.5 years of age (range 2.5–14). Serum creatine phosphokinase (CPK, or creatine kinase, CK) levels were massively elevated along with lactic dehydrogenase and aspartate transaminase. Skeletal muscle troponin-I (STnI) is even more sensitive and specific [148]. Ten children (3%) developed severe rhabdomyolysis, eight had acute renal failure, two required MV, and another one died. The authors referenced other calf muscle biopsy reports in pediatric IAM, demonstrating patchy necrosis with scant inflammatory infiltration in 11 of 12 in one series and 28 of 35 with muscle degeneration, necrosis, and scant infiltrates in another. Because of the lack of infiltration, the authors used the term myopathy in lieu of myositis. All such findings are important because the diaphragm too could be vulnerable to “direct muscle invasion by virus particles or immune-mediated muscle damage.” This could have been responsible for the two cases requiring MV and the one who died. Cell injury along with inflammatory mediators might have contributed to progressive diaphragm fatigue, excitation, and ultimate respiratory failure. Indeed, Eisenhut (2011) [90] reported marked diaphragmatic abnormalities in a 5-month-old girl admitted with RSV bronchiolitis and poor feeding who succumbed in hospital to a sudden, unexpected death. Although grossly normal, diaphragm histology revealed myofiber destruction, focal segmental myocyte necrosis, myocyte regeneration, and focal infiltrates of macrophages and small lymphocytes. Interestingly, similar findings were reported in COVID-19 deaths in adults [47,149]. Such changes provide even more support to evaluate diaphragm histology in sudden death cases involving viral (or bacterial) infection. Moving forward, serum CK, CK-MM (skeletal muscle isoenzyme) or sTnI levels and venous blood gases could screen for and risk-stratify those at risk for respiratory decompensation by critical diaphragm fatigue and excitation.

High CPK levels were also reported in malignant hyperthermia, where there is limb muscle rigidity, spasms, rhabdomyolysis, and myonecrosis caused by various anesthetics. Like Kariks’ diaphragm study, limb histology revealed CBN, segmental necrosis and degenerating muscle fibers caused by “prolonged hypercontraction” [28]. Perhaps by extension, then, some of the deaths in this high mortality condition are caused by respiratory myopathy and Type II failure by DCC. Although no autopsy reports were available examining diaphragm histology in malignant hyperthermia, preceding DD is supported by the author’s mention of “unexplained persistent rises in end-tidal CO_2_ levels”.

**Figure 11:**
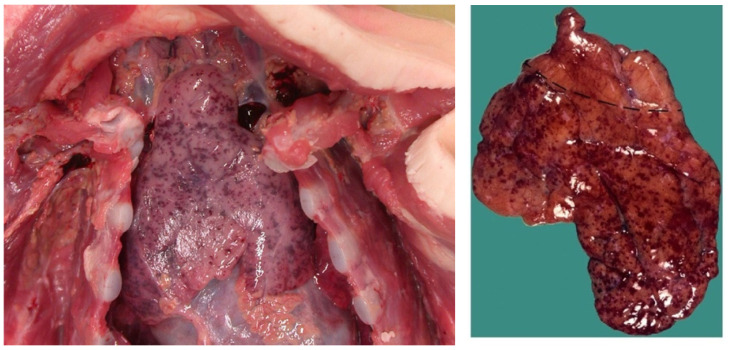
Tardieu petechiae. **Left**: Fetal thymus covered with petechiae. **Right**: Infant thymus showing distinct boundary between the intra- and extrathoracic portions, with and without hemorrhages, respectively (broken line). With permission by source (**left**): © Prof. Paul Goldwater. Source (**right**): © Prof. Roger W. Byard.

Another important finding at autopsy in SIDS and SUDC are intrathoracic Tardieu spots. These are petechial hemorrhages found on the linings of thoracic organs exposed to terminally negative air pressures, such as the epicardium, pleurae, and intrathoracic thymus (Figure 11) [150]. They are present in roughly 80% of SIDS [151] and 50% of SUDC [152] (as well as 30% SUDEP [153]), also seen in septicemia, barotrauma, heat stroke, severe burn injuries, and some electrocutions [154]. Like the Poets study [3], they are thought to occur by agonal breathing against airway obstruction. Again, the cause has never been elucidated, only speculated to be laryngospasm or bronchospasm. The novel airway obstruction of DCC, however, could be it. This also provides a uniting terminal mechanism among the various causes of Tardieu petechiae.

Beckwith’s 1988 paper on Tardieu spots provides an excellent account on their pathogenesis [155]. Two things require mention: (1) vigorous respiratory efforts are required to produce them, and (2) they developed when airway obstruction was induced experimentally at end-expiration and not any other phase of the respiratory cycle. Again, this is consistent with the case patient’s observation of bearhug apnea always being triggered at end-expiration. Perhaps maximal negative intrathoracic pressures are generated then, when DCC obstruction strikes, and tidal volume is fully expelled, thus giving a mechanical advantage to produce the hemorrhages. Moreover, it is just after this phase of the respiratory cycle when diaphragm contractions are first initiated (by neural stimulation). Like limb muscles, this is precisely when cramps are initiated—that is, upon their initial shortening [156]. Future studies to reproduce Tardieu spots might be accomplished by inducing diaphragmatic paralysis in anesthetized infant test animals with bilateral surgical phrenectomies.

A fascinating response arises as to the question of why our patient first experienced DCC at age 7 and not any sooner. The only thing that had changed in his young life was ceasing his lifelong history of nocturnal thumb-sucking. Surprisingly, a literature review revealed several papers, including systematic reviews, supporting the role of pacifiers (dummies) as being SIDS-preventative [118,119]. Perhaps they reduce diaphragmatic workload or minimize ventilatory pausing at end-expiration (when the diaphragm relaxes maximally and about to contract for the next cycle), thus negating the opportunity for DCC to strike. Unfortunately, studies comparing respiratory waveforms both with- and without pacifiers could not be found. This too should be studied, by chest impedance, RIP, EMG and airflow monitoring using sleeping infants as their own controls.

Moving forward, experimentally confirming and reproducing all above pathological findings might be best accomplished by delivering percutaneous electrical currents to the diaphragms of anesthetized test animals. Even better, rebreathing nicotine and exhaled gases in dehydrated, septic, hyperthermic, and acidemic animals might accomplish it. In addition, it would be helpful to look for such changes in nicotine toxicity. This is important not only because of its extreme potency but also the ubiquity of nicotine vaporizer solutions worldwide and thus the cumulatively massive potential for serious harm by unintentional oral overdose, especially in children as they are more vulnerable. Treatment in such cases could start with preventing pathological excitation by reducing DD and workload.

### 2.8. Potential Complications of DCC—Speculation Alert!

**Figure 12:**
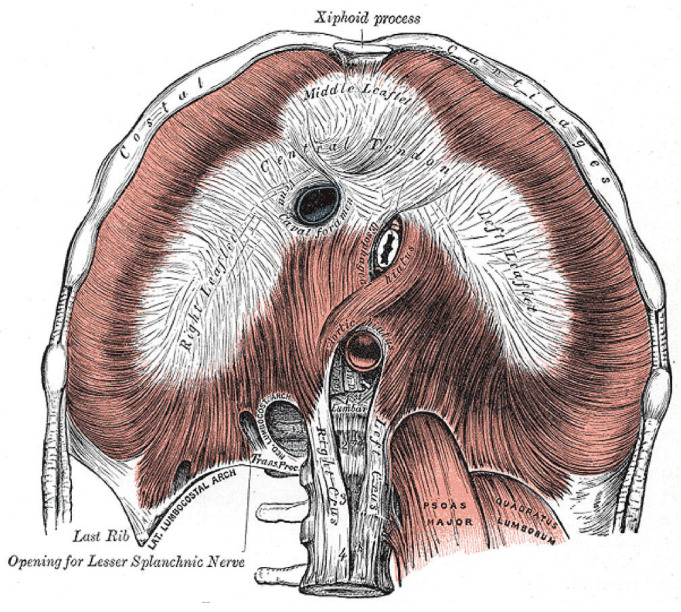
Anatomy of the inferior (abdominal) diaphragm. The diaphragm is composed of tendon and muscle, which tightly wrap around three apertures (hiatuses): the inferior vena cava (IVC), aorta, and esophagus. Diaphragm hypertonicity and pathological excitation could effectively clamp these structures, potentially leading to serious hemodynamic and gastrointestinal consequences both above and below the diaphragm. Courtesy of Henry Vandyke Carter. Public domain, Wikimedia Commons, 2008.

A host of serious pathological consequences might stem from the unique combination of diaphragm anatomy, hypertonicity, and pathological excitation. Nowhere else does this appear in the body. Looking at Figure 12, the musculotendinous diaphragm wraps around three tightly enveloped hiatuses, each providing passage of an important structure: the inferior vena cava (IVC), aorta, and esophagus.

Under increased diaphragmatic tone, spasms, cramps and other DHD, these structures could become squeezed and effectively clamped by the diaphragm itself. Duration and intensity of tone would dictate symptoms, also depending on the form of excitation: from transient in spasms to rhythmic in flutter and sustained in hypertonia and cramp-contracture. Elevated tonic electrical activity of the diaphragm (tonic Edi, i.e., “sustained diaphragm activation throughout expiration”) in 431 PICU patients was associated with the sickest ones in the acute phase of illness, and independently associated with bronchiolitis, tachypnea and hypoxemia [157]. Affecting the youngest patients (0–12 months old versus 1–18 years) and occurring more often in those spontaneously breathing (versus MV), hypertonic Edi also predicted extubation failure. All suggests elevated tonic Edi could be a surrogate of diaphragm fatigue. Support for esophageal clamping is already provided by reports on diaphragm flutter in which a variety of gastrointestinal complaints were made, including hiccups, belching, retching, acid reflux, vomiting, and epigastric (or chest) pain (and visible pulsations) [158]. Similarly, reflux occurs in OSA as mentioned above, and how it improved with CPAP [134]. It could be caused by intermittent esophageal clamping and release from diaphragm spasms combined with negative intrathoracic pressures generated by breathing against the diaphragm. A reduced diaphragmatic workload with CPAP would explain the improvement. Alternatively, GERD in OSA could be secondary to esophageal sphincter weakness or increased gastric acid secretion with decreased salivation and swallowing during sleep. However, none of these would be expected to improve with CPAP. Notably, a medical history of reflux-aspiration is also common in sudden infant deaths [2], supporting the notion of diaphragm hyperexcitation. Also, it is interesting to consider that acute anxiety might physically manifest at the diaphragm. It too could increase tone, leading to the commonly experienced gastrointestinal complaints that are nearly identical to those of diaphragm flutter. But what follows next is even more speculation on a host of potential complications from the hemodynamic-cardiopulmonary standpoint.

As opposed to the aorta, the IVC is thin-walled and under lower vascular pressures, thus more collapsible by clamping. Cardiac preload would drop, leading to reduced stroke volume and cardiac output. Along with aortic clamping and negative intrathoracic air pressure from breathing against obstruction, pulmonary arterial hypertension (PH) and raised hydrostatic pressures in the lungs would develop, resulting in pulmonary congestion and edema. With transient diaphragm spasms, these changes would be reversible, and the patient likely survives (consistent with a diagnosis of noncardiogenic pulmonary edema if substantial). However, with persistent clamping by DCC combined with agonal breathing, the PH and such findings would worsen (also forming Tardieu petechiae). Other associated pathologies from aortic clamping could include simultaneous acute left- and right-sided dilated cardiomyopathies (possibly Takotsubo cardiomyopathy), labile hypertension with hypertensive urgency/emergency and acute heart failure with increased risk of arrhythmias and sudden deaths. Severe hypotension (shock) below the diaphragm would lead to diaphragmatic hypoperfusion, leading to further hypoxemia and diaphragm excitation. This might explain the drastic desaturations observed in neonates with HE. It could also explain the increased risk of necrotizing enterocolitis [122]. Right-to-left cardiac shunting is possible too, perhaps through a patent foramen ovale or ductus arteriosus. This could explain paradoxic motion of the interventricular septum [159] and valvulopathies seen in some patients with OSA [160]. With IVC clamping, hemostasis of the venous circulation inferior to the diaphragm would occur, thereby contributing to venous thrombosis of the lower extremities with raised risk for thromboembolism (VTE) as well as pulmonary embolism and even stroke (paradoxical embolus through patent foramen ovale). Indeed, OSA has been identified as an independent risk factor for VTE (and stroke) [161]. In that paper, the proposed mechanisms (pro-inflammatory state, intermittent hypoxia, and endothelial dysfunction) do not weigh up to the major mechanical hemodynamic changes of potential DHD. 

Lastly, persistent esophageal clamping by agonal DCC would keep stomach contents held under pressure, only to be released and expelled postmortem from diaphragm relaxation. These would collect in the mouth and airways, commonly seen at autopsy in SIDS and other sudden unexpected deaths (e.g., SUDC, SUDEP, malignant hyperthermia) [162]. Alternatively, these findings could be artifacts from passive movements by postmortem body handling (however, CPR was ruled out in an autopsy study [163]).

Evidence supporting the above co-pathologies in DHD was scarce; however, some was provided by elevated pulmonary arterial pressures, as measured by echocardiography and right heart catheterization (gold standard). Transient PH was demonstrated in preterm infants with the onset of HE [35]. It was also implicated in OSA in adults (particularly during REM sleep) [164], SUDEP sheep models [165] and most recently, Type II respiratory failure in an adult with congenital myopathy [166]. In all such cases, PH was idiopathic. Autopsy evidence supporting putative DCC clamping of the IVC in SUDC included brain weights exceeding the 100th percentile in 53 of 56 cases, associated with cerebral edema and vascular congestion [152].

### 2.9. Diaphragm Failure in Bronchiolitis Deaths

Deaths in children with bronchiolitis, primarily from RSV, generally occur by sudden unexpected ventilatory fatigue and failure (terminal apnea) [93,167]. This shares risk factors with SIDS and SUDC, affecting younger or premature infants and those with preexisting respiratory, cardiac or neuromuscular disease. Healthy infants can be affected too, in which apneas strike without preceding signs of infection or respiratory distress. The bronchiolitis occurs from lower respiratory infection typically by community-acquired viral infections that are not fatal. However, lung infiltrates and atelectasis add diaphragmatic workload, where, along with other fatiguing factors similar to above in illness, are proposed to trigger terminal DCC-apnea from a ventilatory workload surge. Alternatively, the apnea could be centrally caused by direct CNS viral infection with resultant cerebral inflammation and edema impairing the respiratory centers [168].

**Figure 13:**
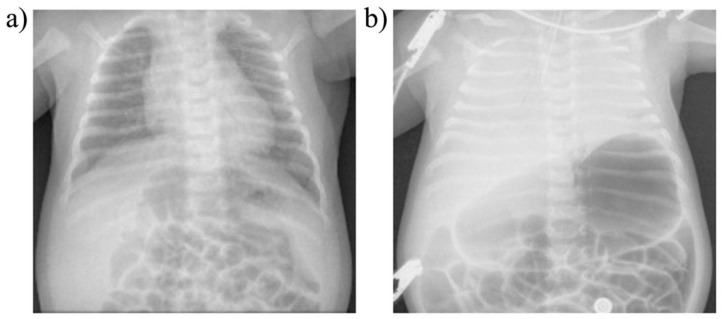
Rapid interval chest X-ray findings in an infant with terminal RSV bronchiolitis. (**a**) Normal-appearing CXR with some streaky lung infiltrates. (**b**) Marked changes 2 h later after sudden respiratory distress, with complete bilateral lung field opacification and ectasia of the stomach and proximal bowels. The opacity is probably from bilateral fluid collections (thought to occur by DCC airway obstruction). Creative Commons Attribution 4.0 International License. Source: Bottino et al. [168].

Figure 13 reveals significant abnormalities on chest X-ray that rapidly developed in a 40-day-old infant admitted with RSV. The baby, who had presented with fever, cough and wheezing, suddenly deteriorated with respiratory distress marked by hypoxemia, hypercapnia and respiratory acidosis. Terminal apnea and death occurred before intubation could be done. Complete opacification of both lung fields with an air-dilated stomach and proximal bowels developed in less than two hours. No explanation could be provided. Although diaphragm pathology was not reported, the clinical findings and X-ray changes were consistent with progressive diaphragm fatigue (insufficiency), sudden failure (respiratory arrest) and agonal airway obstruction by DCC. Terminal ineffective RAM efforts could have led to substantially negative intrathoracic pressures with resultant pulmonary shunting, edema, hemorrhage, and fluid extravasation. Also, intrapulmonary air could have been forced into the digestive tract, perhaps trapped by the putative esophageal hiatus clamp, leading to reduced pulmonary expansion from increased intra-abdominal pressure. By contrast, in MV patients, such findings have not been reported to our knowledge. This could be due to the reduced work of breathing averting DCC obstruction. 

### 2.10. Diaphragm Failure in Seizure Deaths (SUDEP)

There are several overlapping historical and autopsy findings among SIDS, SUDC and SUDEP, and this suggests a common pathological mechanism. We believe this to be DCC. SUDEP typically affects young adults who suddenly expire silently, unwitnessed at night, probably while sleeping, only to be found afterwards without overt signs of distress in prone bed position with aspirated gastric contents in mouth and airways. The lungs are very “wet” and heavy, engorged with blood and fluid. Tardieu spots are not uncommon (at least 30% of cases). These findings are in keeping with those of Zhang et al. (2022) [153], who, using controls, determined at autopsy the primary mechanism of death in 13 SUDEP cases was asphyxiation (Tardieu spots, pulmonary congestion, and hemorrhages). Those in prone position were at significantly higher risk. Generally though, other than this, autopsies in SUDEP are considered “negative” (but yet again, diaphragm histology is not being done).

**Figure 14:**
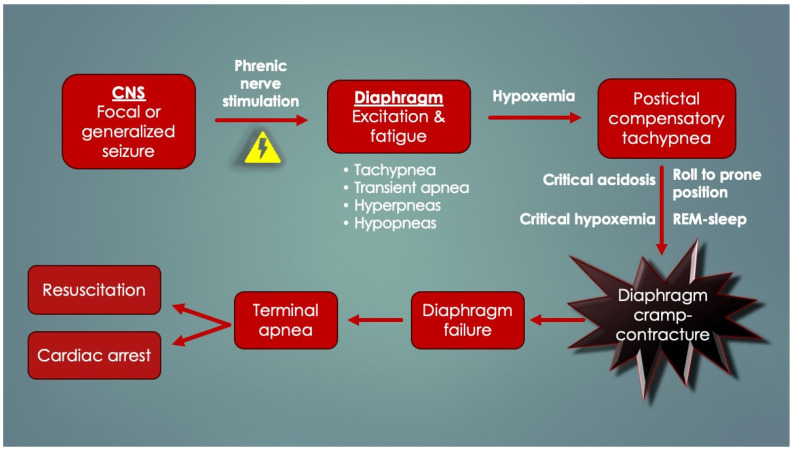
Postictal diaphragm cramp-contracture hypothesis in seizure deaths. This differs from other SUDEP hypotheses by (1) seizure-induced diaphragm excitation (and fatigue) carried by the phrenic nerve(s), (2) worsened fatigue from postictal tachypnea and (3) sudden excitation by DCC, leading to silent terminal apnea and cardiac arrest.

Zhang’s findings are supported by Ryvlin’s MORTEMUS study (2013) [169], which elucidated terminal events in fatal seizures by retrospectively assessing patients who had continuous EEG, video and basic cardiorespiratory observations (respiratory movements) performed and recorded at epilepsy monitoring units. In ten confirmed SUDEP cases, postictal tachypnea occurred in all, followed by a terminal apnea and cardiac arrest either within three minutes or delayed by 11 min after temporary restoration of cardiorespiratory function. Over 90% had died in the prone position. This redirected attention from the heart to the respiratory system and CNS in SUDEP. Most of the seizures had started in the temporal lobe, a region involved in volitional breathing. Although the precise cause could not be elucidated (no airflow, chest impedance, oxygen saturations or end-tidal carbon dioxide levels), the terminal apnea was speculated to be centrally mediated by “post-generalized EEG suppression”. However, this is at odds with the asphyxia of the Zhang study and its autopsy findings. Instead, periictal diaphragm excitation and consequent fatigue could have occurred by *seizure transmission along the phrenic nerves*. Seizure spread to a peripheral nerve (recurrent laryngeal), causing end-organ (laryngeal) spasm and respiratory arrest is not a unique idea, as it was performed experimentally in rats [103]. Intracellular and systemic lactate, a byproduct of seizure causing metabolic acidosis, could have developed over time, thereby contributing to progressive lengthening of diaphragmatic T_R_, worsened by the postictal (compensatory) tachypnea. This is consistent with the delayed aspect of some deaths. Postictal diaphragm fatigue exacerbated by prone positioning, REM sleep, progressive hypoxemia, and/or critical acidosis could have triggered terminal DCC-apnea. Given the silent internal struggle of the obstruction, this would not have easily been picked up by the cardiorespiratory observers. The general scheme for DCC in SUDEP is depicted in Figure 14.

It is important for the DCC hypothesis to explain the rarity of deaths from SUDEP compared to the overall number of nonfatal seizures in the population. Table 4 lists potential factors contributing to survival versus mortality in seizures from a hypothetical DCC standpoint. Important *fatality* factors by immediate or postictal DCC include (1) hyperstimulation of the respiratory muscles (may not always be the case in seizures); (2) bilateral diaphragm hyperstimulation is followed by bilateral excitation in DCC, leading to complete bilateral diaphragm paralysis (some seizures are unilateral); (3) preexisting diaphragm fatigue, if it occurs, is worsened by the seizure (i.e., variable contributions by prone positioning, nicotine exposure, or intercurrent infections with dehydration and diaphragm myositis); and (4) sudden postictal RAM inactivation by REM sleep or excitation (RAM spasms or cramps). *Survival* factors include (1) subcritical seizure duration (minimal postictal metabolic derangements like lactic acidosis and overheating), (2) subcritical hypoxemia (no cardiac arrest), and (3) postictal diaphragm spasms that are transient and recoverable but not persistent, like DCC.

Diaphragm involvement in fatal seizures is supported by a SUDEP mouse EMG study in which all deaths also occurred by terminal apnea. They carried mutations of a sodium ion channel protein (*Scn8a*) identified in SUDEP victims. The channel is expressed in both sensory and motor neurons of the central and peripheral nervous systems. By inducing seizures while measuring D-EMG, Wenker et al. (2021) [115] discovered terminal apneas occurred by continuous (tonic) diaphragm contractions. Deaths did not occur in those receiving MV. Perhaps it was life-sparing because of a reduced work of breathing (no DCC), or if excitation had occurred, MV overcame the airway resistance of diaphragm hypercontraction.

A final study supporting diaphragm excitation in seizure emerges from a study of 100 children under twelve years old with partial epilepsy using videotaped seizure analysis and data collection during the preictal, ictal, and postictal phases [117]. Again, most of the 514 seizures were localized to the temporal lobe. The entire array of presentations in descending order of frequency included flushing, coughing, apnea*/bradypnea*, epigastric aura*, hyperventilation*, dyspnea*, hypersalivation, vomiting*/nausea, spitting, miosis, hiccups* and belching*. Although assumed secondary to autonomic causes, nearly all could have rather related to the diaphragm itself (marked by “*”). In other words, seizure activity could have been carried by the phrenic nerves, causing diaphragm excitation and fatigue, followed by spasms and gastroesophageal clamping. This is supported by a case report of a 6-year-old girl with daily hiccups associated with bilateral myoclonic D-EMG bursts following epileptiform EEG activity [170]. Symptoms resolved with valproate. Epigastric, or visceral, aura is a symptom complex of short duration involving ictal abdominal discomfort, nausea and/or a burning sensation. It occurs most often with temporal lobe seizures and receives unusual descriptions such as “fluttering, pressure and rolling or turning of internal organs”. Again, this is consistent with putative diaphragmatic “butterflies” and other mild DHD symptoms. However, extreme fear and panic sometimes occur, and perhaps this is not without good reason. Sudden diaphragmatic inactivation by a focal seizure in a child, essentially a broken pump causing inability to breathe in the face of rapidly escalating hypoxemia and hypercapnia, would most certainly trigger severe apprehension, panic and an impending sense of doom. Interestingly, such symptoms also occur in abdominal winding injuries.

### 2.11. Diaphragm Spasm and Cramp in Winding Injuries

**Figure 15:**
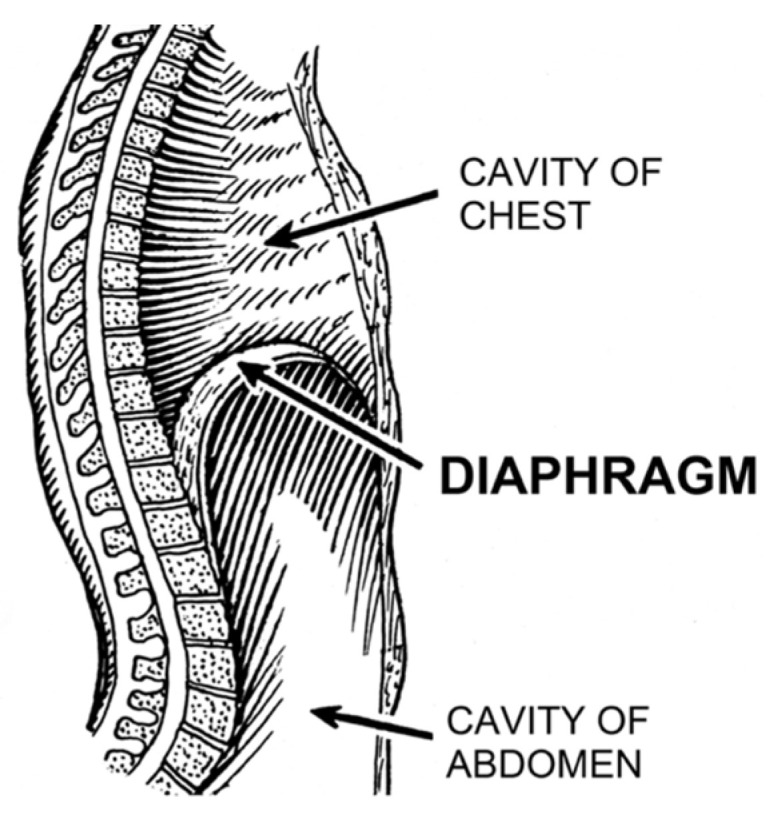
Diaphragm anatomy in relation to chest and abdomen (lateral view). The respiratory diaphragm, a hermetic seal like the tympanic membrane of the ear, separates the chest from abdominal cavities. A blunt blow to one cavity is transmitted through the diaphragm to the other. The diaphragm can rupture in high-velocity trauma, but with lesser forces (winding injuries), neuromuscular excitation is thought to occur by diaphragm spasm or persistent cramp. Source: Pearson Scott Foresman, public domain, via Wikimedia Commons, 2008.

Like the tympanic membrane of the ear, the diaphragm serves as a hermetic seal between two anatomical compartments under differential air pressures (chest and abdominal cavities) (Figure 15). Both seals can rupture from sudden changes in air pressure: the tympanic membrane in SCUBA barotrauma for example, and the diaphragm in high-velocity blunt impacts to the chest or abdomen (e.g., motor vehicle collisions or falls from great heights). Being winded (celiac or solar plexus syndrome) occurs by a lower velocity blunt blow. It can happen with a punch or kick, a slip and fall onto the back or sometimes, running collisions between NFL football players, one of which did not brace himself in time (Damar Hamlin “cardiac arrest”). Upon a literature review, of which only two old medical textbooks and one reputable web page could be found, diaphragmatic spasm was mentioned to cause “momentary respiratory paralysis” [64,171]. No supporting evidence was available unfortunately.

Interestingly, tensor tympani syndrome is a disorder of the middle ear similar to spontaneous diaphragm spasms. Involuntary myoclonic contractions of this small, striated muscle cause a soft clicking or thumping sound as well as hyperacusis and otalgia. And like diaphragm flutter, it can be triggered by anxiety.

Upon chest or epigastric impacts in winding injury, kinetic forces could be transmitted to the diaphragm. Although there is immediate pain, the worrisome symptom is the involuntary expiration with sustained apnea (forced-expiration inspiratory arrest like some infantile HE). Usually this lasts just a few seconds. However, as above (epigastric aura in focal seizures), the commonly experienced sense of impending doom is perhaps not without good reason. Although most winding injuries are short-lived and seemingly benign (from putative transient diaphragm spasms), it is conceivable that higher-force injuries could be fatal from prolonged apnea (respiratory arrest by persistent spasm, or “traumatic DCC”). This would be followed by hypoxemic syncope and possibly seizure as well as cardiac arrest and death if not rapidly aborted. Such fatalities would go on to be diagnosed postmortem as traumatic cardiac arrests or commotio cordis, thus missing the primary respiratory pathology. Commotio cordis typically occurs by a projectile, like a baseball or hockey puck, striking the left chest over the heart and inducing ventricular fibrillation and cardiac arrest. This may not necessarily have been the case in the Hamlin collision though (by assuming forces were transmitted to the heart yet without the focal aspect defined by commotio cordis experiments) [172]. Importantly, CPR priorities differ between the two: cardiac compressions in commotio cordis versus rescue breathing in winding injury arrests.

In summary, the kinetic force of blunt trauma transmitted to the diaphragm could trigger neuromuscular excitation in the form of spasms and cramps. Apnea results and its duration would dictate survivability based on the degree of subsequent hypoxemia and potential for cardiac arrest. Moving forward, reproducing winding injuries under experimental conditions in test animals using fluoroscopy could reveal the traumatic forms of DHD.

### 2.12. Diagnosing Diaphragm Fatigue and Excitation in Children

Objective signs of diaphragm fatigue in infants are those of respiratory insufficiency, including tachypnea and laboured breathing, grunting, wheezing, sweating and rib and subcostal retractions. A suddenly silent chest and paradoxic breathing are late findings. From frequent hiccups and respiratory instability in neonates, to bronchiolitis, apneas, breath-holding spells or life-threatening symptoms in older babies and children (with cyanosis, pallor, hypotonia, altered mental status or brief resolved unexplained events), the various etiologies and parameters of underlying diaphragm fatigue can be evaluated (Table 5 and Table 6). This includes labs to rule out anemia, electrolyte disorders and abnormal acid-base balance. Screening tests to reveal diaphragm damage from hypoxia, hyperthermia and myopathic viral infections include serum CK, CK-MM and STnI. Although not specific to respiratory muscles, correlating them to changes in clinical state would be helpful. In bronchiolitis, these might screen for those at risk for apneas. Those with elevated levels should be admitted for respiratory monitoring by chest impedance or, even better, continuous RIP and transcutaneous D-EMG. In those with respiratory distress, bedside abdominal ultrasound might reveal the supraphysiologic excursions of DHD (i.e., spasms, flutter, cramps). Video fluoroscopy would also provide direct visual evidence of DHD and could be done bedside using a “C-arm.” Dynamic chest radiography, a newer technology, offers a better alternative to fluoroscopy because of lower exposure to ionizing radiation. Of special mention, electrical activity of the diaphragm, specifically, high tonic Edi (and prolonged duration into expiration), appears to reflect diaphragm fatigue as discussed above [157]. It might predict respiratory deterioration. D-EMG by esophageal catheter is superior to transcutaneous due to fewer artifacts from noise and movement. Finally, diaphragm biopsy could be reserved for cases with severe respiratory insufficiency when the diagnosis of diaphragm weakness in neuromuscular disease or DHD are in question. Diaphragm histology could reveal evidence of infectious myositis and myopathy as well as scarring from repeated HE.

### 2.13. Treating Diaphragm Fatigue and Excitation in All Ages

Table 7 provides emergent interventions and potential medications identified on preliminary literature review that might improve DD and thus prevent or attenuate excitation. In patients of any age with respiratory distress or infants with ventilatory instability, this includes correcting anemia, electrolyte disorders and acidosis as well as providing supplemental oxygen, rehydration and antipyretics as needed. In addition, it is prudent to stop anti-reflux medications until electrolyte levels are confirmed normal (i.e., sodium, potassium, calcium, magnesium, phosphate). Methylxanthines such as caffeine and theophylline have been used for over 50 years to reduce apneas-hypopneas and HE in infants. These are thought to stimulate the CNS respiratory centers; however, also have peripheral effects at the respiratory muscles [132,173]. Chlorpromazine, an older antipsychotic dopamine antagonist with relaxation effects on the CNS and skeletal muscles, was most commonly used to treat DHD, particularly effective in intractable hiccups and respiratory flutter [13]. Its calming effect might even reduce diaphragmatic hypertonicity in extreme anxiety. N-acetylcysteine has shown promise by improving diaphragm force-generating capacity and anti-inflammatory activity in a mouse model of muscular dystrophy [174]. Finally, diaphragm pacing might treat or avert unstable DHD.

Treatment is a high priority in OSA because of its ubiquity and serious comorbidities. But despite use of positive airway pressure masks improving OSA severity in adults (i.e., CPAP and nasal masks), long-term benefits such as reduced hypertension were minimal [175]. Instituting therapies tailored to reducing respiratory muscle fatigue and workload in OSA offers a novel approach.

### 2.14. Diaphragm Pathology in Decompensating Medical Conditions and Cardiopulmonary Arrest

There are a wide variety of emergent medical conditions appearing to share in common terminal respiratory decompensation and sudden arrest consistent with DD and DCC, respectively. Many have already been discussed. Recognizing this, especially in “matter of life and death” situations, offers an opportunity for clinicians to immediately institute treatments that could improve ventilatory muscle function. Table S2 lists these conditions along with supporting citations. Some are provided here.

Well-known chronic illnesses terminating in end-stage respiratory distress include a wide variety of neurologic and myopathic diseases, such as amyotrophic lateral sclerosis, myasthenia gravis and muscular dystrophy. Other emergent conditions are asthma, COPD, pneumonia and sepsis as well as bronchiolitis and severe gastroenteritis dehydration in children (hypovolemic shock with acidosis). Other shock states may include hemorrhagic, neurogenic and cardiogenic (the latter by myocardial ischemia, myocarditis, arrhythmias, pulmonary embolism or cardiac tamponade for example). Survival could also be improved in severe acidosis, such as diabetic, starvation and alcohol ketoacidosis as well as metformin lactic acidosis and toxicities from methanol, ethanol and ethylene glycol. Less common conditions include respiratory complications of malignant hyperthermia, anesthesia induction, pheochromocytoma crisis, tetanus, rabies and malnourishment. Traumatic examples include diaphragm weakness from birth asphyxia and phrenic nerve injuries, as well as severe winding injuries, traumatic and restraint cardiac arrests and crush syndrome. Exposures include heat stroke, cold water near-deaths (near-drownings and immersions) and electrocutions, including conducted electrical devices (tasers and stun guns). Finally inclusive are those surviving severe seizures.

To best combat the respiratory arrest of DCC, CPR guidelines for both healthcare professionals and the community need to be revisited. In all pediatric and winding injury cardiopulmonary arrests, life support measures should first focus on the airway and breathing before chest compressions, given the cause is almost always a respiratory emergency. Education should stress the importance of opening and maintaining the airway and administering effective rescue breaths by visually confirming chest rise (not explicit in the current guidelines). Also essential is to overcome the initial airway resistance of DCC and not fear overinflating the lungs until this is accomplished.

## 3. Conclusions

It is exceptional the wealth of information that stemmed from only two symptoms in a solitary patient, let alone one who survived their repeated near-death experiences starting when just 7 years old while in bed alone. It appears he overcame the paroxysmal bearhug apnea episodes, deduced to be spontaneous diaphragm cramps, because his wherewithal and troubleshooting ultimately led to impromptu rescue breaths that overpowered the tension of muscle contracture. Literature evidence for novel DCC in sudden unexpected deaths is highly compelling, albeit indirect for the time-being, as this mechanism of silent respiratory arrest hides from sight both in vivo and postmortem.

Some of the best support was provided by EMG studies in preterm infants with apneas, demonstrating simultaneous failure of diaphragm and RAM (Lopes et al), and in adult sleep apneas, abnormal diaphragm electromechanical activity (dissociation) (Luo et al). Dr. Eisenhut’s evidence of diaphragm myositis-myopathy in an infant with RSV respiratory arrest was also quite significant. Siren and Siren’s papers helped build the foundation supporting DCC as a complication of critical diaphragm fatigue. Case reports of persistent hiccups, diaphragm spasms and flutter revealed the existence of various hyperexcitable disorders (DHD), many of which were triggered by psychological distress amongst other factors. Moreover, they presented along a novel frequency spectrum correlated to worsening symptoms and prognosis. Critically unstable DHD included high frequency, tetanic contractions of nicotine toxicity and certain electrocutions. Along with DCC, these were proposed to occupy the severe end of the spectrum.

In general, sudden inspiratory arrests by acute diaphragm paralysis are an underrecognized cause of serious morbidity and mortality in all ages. If the apnea is unrecognized in adults, they would be misclassified as sudden cardiac deaths. The paralysis, which is already known to occur with nicotine, succinylcholine and bilateral phrenic nerve injuries, for example, can be immediately fatal when it is bilateral, neurologically complete and affects those with weak or inactivated accessory muscles. Also, it needs to be sufficiently long to induce cardiac arrest by critical hypoxemia. This too was a novel finding that could be responsible for some SIDS cases, because DCC satisfies all such criteria. Optimistically, this provides a window of opportunity in sleeping infants to recognize DCC by improving respiratory monitoring techniques and rapidly instituting ventilatory assistance.

Diaphragm fatigue and excitation are central to the DCC hypothesis in SIDS (as well as non-arrhythmogenic sudden unexplained deaths in all other ages). Diaphragm fatigue, the outcome of mismatched metabolic supply and demand—which appears to occur more often in males than females—is exacerbated by hypoxemia and numerous other endogenous and exogenous factors that generally impair diaphragm contractility, prolong its relaxation or increase ventilatory workload. These overlap with most, if not all, SIDS risk factors. Endogenous ones include male sex, prematurity-young infancy, prone position, REM sleep RAM inactivation and upper airway obstructions as well as fever, sweating, pneumonia, bronchiolitis, bronchospasm, dehydration, electrolyte disorders, acidosis, anemia and seizures. Exogenous factors include nicotine, over-bundling, rebreathing, viral infections and bacterial toxins. Consequent diaphragm insufficiency reduces alveolar ventilation. This perpetuates the hypoxemia (hypercapnia and respiratory acidosis as well), leading to escalating fatigue in an unstable, positive feedback cycle. Simultaneously, anemia and diaphragm hypoperfusion from dehydration or hypotension would exacerbate the DD because of a reduced delivery of oxygen and metabolic substrates. In experimental septic and cardiogenic shock in spontaneously breathing animals, hypotension led to fatal, rapidly progressive diaphragm fatigue abruptly terminating in respiratory failure. Although the terminal mechanism could not be elucidated, it was reasoned most likely due to “failure of the contractile machinery”. These deaths were all consistent with DCC (and SIDS).

Prolonged diaphragm relaxation by fatigue, ryanodine, acidosis and endotoxins predisposed to neuromuscular irritability in the form of diaphragm contractures ex vivo. By extension in vivo, diaphragm fasciculations, spasms, cramps and flutter could develop, particularly under higher respiratory rates when relaxation takes longer than the expiration phase of the ventilatory cycle. Excitation in fatigued skeletal muscles is known to be triggered by workload surges. In sleeping infants, this could occur in the diaphragm by a roll to prone position, REM-sleep onset, bottle feeding or even psychological distress. This could tip the balance from critical fatigue to failure by DCC.

Many of the breathing issues common to preterm infants, such as apneas-hypopneas, hypoxemic-cyanotic episodes, periodic breathing and even frequent hiccups, can all be explained from a diaphragm excitation standpoint (rather than abdominal muscle contractions or CNS dysfunction). The same can be said of OSA in all ages. The novel obstruction of OSA, proposed here, develops when RAM contractions to breathe are resisted by the temporarily inactivated, hypercontracted diaphragm of spasm. Airway obstructions from atonic supraglottic muscles would only contribute because of the added fatigue.

At the histological level in SIDS at autopsy as well as rapid deaths in RSV bronchiolitis, diaphragmatic myositis-myopathic changes already provide a starting point to uncover evidence of the anoxic hypercontraction injury thought to be DCC itself. Therefore, *diaphragm histology needs to be done at autopsy*. Critical acidosis and extreme hyperkalemia were reported in SIDS but never corroborated. Along with other electrolyte disorders, these would predispose to DCC through their various pathological effects on excitation-contraction coupling.

In adults with OSA, putative diaphragm hypertonicity-excitation-clamping of the IVC and aorta could explain many of its known complications, such as pulmonary and systemic hypertension, cardiomyopathies, pulmonary edema and a host of other serious cardiovascular disorders. In spasms, such acute changes would be transient and modest, whereas in flutter or DCC, more persistent and severe. Increased diaphragmatic tone in acute anxiety, if confirmed, would represent a novel connection between mind and body. Epigastric “butterflies” could be such a manifestation. Moreover, esophageal clamping would explain the host of gastrointestinal symptoms common to anxiety, also reported in diaphragm flutter and even some pediatric temporal lobe seizures. In the latter, we proposed seizure activity transmitted by the phrenic nerves hyperstimulated the diaphragm, causing periictal hyperpneas and hypopneas-apneas leading to net hypoxemia. Subsequent diaphragm fatigue would be worsened by prone positioning and postictal compensatory tachypnea. Critical hypoxemia, hyperacute acidosis or REM sleep could be the final trigger leading to DCC respiratory arrest, ultimately causing a rapid, silent death consistent with SUDEP. Finally, winding injuries appear to induce forced-expiration inspiratory arrests by diaphragm spasm or DCC, depending on severity. Like infantile expiratory apneas, the former is thought to be transient whereas the latter, persistent. Apnea duration would dictate degree of hypoxemia and risk for syncope, secondary cardiac arrest and death. The mechanism and ensuing collapse would mimic commotio cordis and other traumatic cardiac arrests and is important for future studies to distinguish because their CPR priorities differ.

Astoundingly, thumb-sucking appeared to have spared our case patient’s life (as his DCC only started after ceasing the nocturnal habit). Pacifiers (and likely digit-sucking), which are known to be SIDS preventative, might reduce diaphragmatic workload and minimize ventilatory pausing at end-expiration, when excitation appears to strike. Autoresuscitation in the case patient apparently occurred by breathing out to breathe in, augmented by positive-pressure inspirations. CPR in DCC respiratory arrests needs to focus on maintaining a patent airway and confirming adequate rescue breaths while overcoming the initial airway resistance.

Screening labs for diaphragm injury and excitation include venous gases and CK-MM or sTnI, although these are not specific to respiratory muscles. Regardless, they might risk-stratify infants with RSV and other viral infections for prolonged apneas. The same would apply to former preterm infants about to be discharged from the neonatal ICU. Bedside tests in active DHD include diaphragm ultrasound and fluoroscopy. Respiratory monitoring could be accomplished by continuous RIP or D-EMG. Therapeutics for diaphragm fatigue include caffeine, CPAP and chlorpromazine amongst others. The latter offers a novel approach to prevent and treat respiratory instability in infants as well as OSA in all other ages. Diaphragm pacing might avert unstable diaphragmatic arrhythmias in high-risk individuals. Above all else however, *DCC needs to be confirmed and reproduced experimentally*.

**Figure 16:**
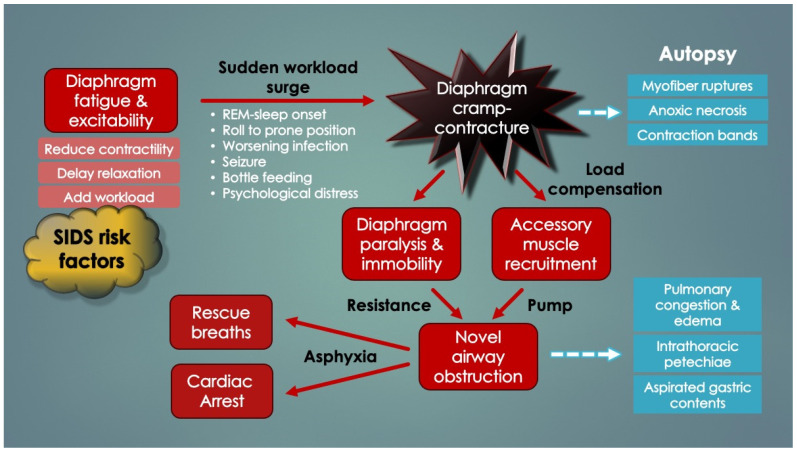
Proposed diaphragm cramp-respiratory arrest pathogenesis in infants. Most, if not all, SIDS risk factors fatigue the diaphragm, increase its workload or delay relaxation. Increased tonicity and excitation develop in skeletal muscles when fatigued, and respiratory muscles like the diaphragm are likely not exempt. Diaphragm cramp is thought to be triggered by ventilatory work overload. A novel airway obstruction would develop where the paralyzed diaphragm resists inspiratory efforts by agonal RAM contractions. Postmortem findings consistent with DCC include disrupted myofibers and anoxic contraction band necrosis (both of which are visible only microscopically) as well as signs of asphyxia by terminal airway obstruction. Respiratory arrest can be reversed by rescue breaths, but the window of opportunity is short given cardiac arrest will ensue within minutes.

**Figure 17:**
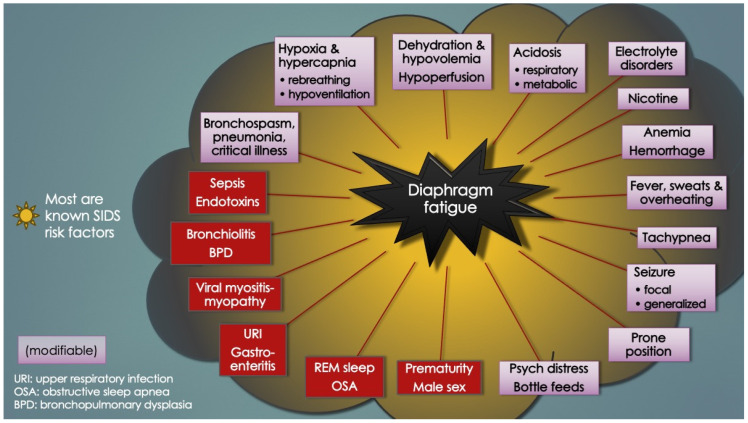
Diaphragm fatigue/excitation factors. These overlap with known SIDS risk factors. Some are modifiable, like giving oral fluids for dehydration, blood transfusions in anemic apnea and avoiding nicotine exposure.

Figure 16 depicts the overall DCC pathogenesis in SIDS. Figure 17 demonstrates the numerous causes of diaphragm fatigue discussed here. The Patient’s Perspective and supplementary tables, figures and video are also available.

## Figures and Tables

**Table 1:** Etiologies of acute and chronic respiratory muscle paralysis and paresis leading to Type II hypercapnic failure. The CNS, phrenic nerve(s), diaphragm, and/or RAM may be involved. Insults can be sudden or gradual in onset, bilateral or unilateral, and neurologically complete (paralysis) or incomplete (paresis). Diaphragm fatigue, that is, temporary weakness reversed by rest, worsens the clinical course for most chronic conditions. Mortality typically occurs by acute respiratory failure. Diaphragm failure is an underrecognized cause of mortality in all ages.

Multi-Organ (CNS, Phrenic Nerve(s), Diaphragm, Respiratory Accessory Muscles)			Onset	Side	RAM?	Citation
	Electrocution	Lightning, low- and high-voltage shocks	S	Both	✓	[23,24]
	Neurotoxin	Nicotine, botulism, tetanus, curare, organophosphates, carbamates, tetrodotoxin, strychnine, envenomations	S	B	✓	[19,20,37]
	Medication	Neuromuscular blockers, aminoglycosides, catecholamines	S	B	✓	[38,39,40]
	Electrolyte	Hypomagnesemia, hypocalcemia, low and high potassium, hypophosphatemia	S,G	B	✓	[41,42,43,44]
	Metabolic	Acidosis *(DKA)*, endocrinopathies (*pheochromocytoma crisis), eating disorders*	S,G	B	✓	[25,40,45]
	Inflammatory	Vasculitis, pneumonia, pleurisy, herpes zoster, SARS-CoV-2 (COVID-19)	G	B	✓	[29,46,47]
	Neurologic & Myopathic Disease	Guillain-Barré syndrome, polio, ALS, myasthenia gravis, Lyme disease, rabies, muscular dystrophy, polymyositis, dermatomyositis, inclusion body myositis	G	B	✓	[29,46,48,49]
**Phrenic Nerves and Nerve Roots**						
	Traumatic	Cervical spinal cord transections or contusions (above C5)	S	Both	✓	[50,51]
		Phrenic nerve injuries (blunt, penetrating, traction, compression)	S	Both	0	[52,53,54]
	Iatrogenic	Birth trauma (asphyxia), chiropractic manipulations	S	Both	0	[55,56,57,58]
		Cardiothoracic surgeries, cardiac cryoablation	S	Both	0	[59,60,61]
	Compression	Cervical osteoarthritis, tumours (bronchogenic, mediastinal), aortic aneurysm	G	U	0	[29,31,46]
**Diaphragm**						
	Traumatic	High velocity: contusion, hemorrhage, rupture, paralysis	S	Both	0	[62,63]
		Low velocity: *winding injury* (celiac or solar plexus syndrome)	S	Both	0	[64,65,66]
	Asphyxia	*Restraint cardiac arrests, crush injury, chemical asphyxiants*	S	Both	✓	[67,68,69]
	Exposures	Cold water submersion, heat stroke, conducted electrical devices	S	B	✓	[70,71,72,73]
	Spontaneous	*Diaphragm cramp-contracture*	S	B	0	[1]

RAM: respiratory accessory muscles, S: sudden, G: gradual, Both: bilateral and unilateral, B: bilateral, U: unilateral, CNS: central nervous system, DKA: diabetic ketoacidosis, ALS: amyotrophic lateral sclerosis, *Italics:* putative (unproven).

**Table 2:** Diaphragm workload- and fatigue/excitation factors in infants. The fatigued diaphragm is prone to neuromuscular excitation by work overload from a variety of endogenous and exogenous causes. Many are SIDS risk factors. Premature, smaller infants have incompletely developed respiratory muscles as well as less fatigue-resistant diaphragm myofibers. (A) Higher ventilatory workloads occur in neonates than older infants because of a collapsible, unstable chest wall. Ultrasound evidence suggests the male diaphragm works harder and fatigues easier than in females (vide infra). Prone positioning, REM sleep, obstructive sleep apnea (reduced supraglottic patency), and respiratory infections all add workload as does anemia of infancy by a reduced blood oxygen-carrying capacity. Bottle feeding and psychological distress are known to trigger hypoxemic episodes. Pacifiers are SIDS-preventative, possibly digit-sucking too. Both might reduce workload. (B) Nicotine induces skeletal muscle contractures ex vivo, whereas an overdose causes fatal respiratory arrests by diaphragm paralysis. Similarly, fever, acidosis and loss of hydration and electrolytes all impair contractility and delay muscle relaxation, leading to diaphragm fatigue, increased tone and excitability. In some children with RSV or influenza-B calf myopathy, the diaphragm might be affected too (viral myositis-myopathy). Bacterial toxins in sepsis directly impair diaphragm contractility and delay relaxation, leading to fatal respiratory arrests. Hypoxia, hypercapnia and respiratory acidosis from CO_2_ rebreathing and hypoventilation reduce contractility and relaxation. Hypercapnia and hypoxemia both reduce alveolar ventilation, thereby exacerbating both conditions in positive feedback cycles. Seizure-induced tonic diaphragm excitation carried from CNS to phrenic nerves induces diaphragm fatigue terminating in diaphragm arrest apnea in mice.

(A) Increased Diaphragm Workload	Citation	(B) Diaphragm Fatigue/Excitation Factors	Citation
Prematurity/low birth weight (underdeveloped, weak RAM)	[75]	Prematurity/low birth weight (reduced fatigue-resistant, Type 1 myofibers)	[75]
Compliant ribcage (distortion)	[75]	Household tobacco smoke (nicotine)	[19,20,76]
Male gender	[55,77,78,79,80]	Overheating & diaphoresis	[81,82,83,84]
Prone position	[8,85]	Infection (URI, gastroenteritis, bacterial)	[8,86]
REM-sleep RAM inactivation & OSA	[4,8,87,88]	Viremia, bacterial toxins, skeletal muscle myositis-myopathy	[49,89,90,91,92]
Bronchiolitis & pneumonia (atelectasis, infiltrates)	[93,94]	Septic shock	[74,95,96]
Bronchopulmonary dysplasia	[97,98,99]	Metabolic acidosis (tissue hypoxia, hypovolemia, stool bicarbonate loss)	[25,43,100,101]
Laryngospasm, bronchospasm	[102,103]	Rebreathing exhaled gases, hypoventilation	[8,104,105,106,107]
Dehydration, hypotension, shock	[74,108,109,110,111]	Hypoxemia	
Anemia of infancy, hemorrhage	[112,113,114]	Hypercapnia	
Bottle feeding	[33,34,35]	Respiratory acidosis	
Psychological distress, pain	[17,34,35]	Seizures (focal & generalized)	[92,115,116,117]
*Pacifier weaning?*	[118,119]		

RAM: respiratory accessory muscles, OSA: obstructive sleep apnea, URI: upper respiratory infection, Italics: putative (unproven).

**Table 3:** Sequence of at-home respiratory arrest from DCC in an infant. This is a hypothetical example of an otherwise healthy 3-month-old with low-grade fevers, rhinorrhea, congestion, cough, and loose stools from a typical viral infection over the past 24 h. He is sleeping alone in a crib upstairs in a smoking, heated household in winter. Heart rate, oxygen saturations, and respiratory movements (but not airflow) are being wirelessly monitored using a typical home SIDS device. Progressive diaphragm fatigue terminating in pathological excitation by DCC with resultant novel airway obstruction is central to this SIDS hypothesis.

1	Gradually progressive DD, hypoxemia and hypercapnia occur by cumulative effects of prone sleeping (causing rebreathing and a higher diaphragm workload), viral diaphragm myositis and nicotine absorption from cigarette *smoke* (in an *upstairs* bedroom of a *heated* household) *.
2	Fluid losses from fever, decreased oral intake, sweating and bicarbonate-rich diarrhea over past 24 h. Along with overheating and further rebreathing from loose, heavy bed blankets; sweating, dehydration and hyperthermia develop, adding workload. Further hypoxemia, hypercapnia and metabolic and respiratory acidosis ensue, all exacerbating DD.
3	Compensatory RAM recruitment while awake (respiratory load sharing). Inspiratory intercostal muscles activated. Observed bedside as rib retractions.
4	Like fatigued limb muscles, diaphragm hypertonicity develops.
5	Infant falls asleep. REM-sleep inactivation of RAM by CNS. Sudden workload placed on fatigued diaphragm putting it past cramp threshold. Precipitates the painful bearhug of DCC that paralyzes the diaphragm and induces diaphragm arrest.
6	Oxygen saturations slowly begin to drop at first yet insufficient to trigger alarm.
7	Infant wakes from the cramp or critical hypoxemia but with ineffective gasping (inspiratory arrest), is unable to cry out. RAM reactivated with waking.
8	Independent of diaphragm, RAM contract in effort to expand ribcage and lungs to inspire. Met with combined resistance of pulmonary compliance and the hypercontracted, immobilized diaphragm. Like breathing against a 100% upper airway obstruction, the agonal efforts build negative intrathoracic pressures (yet insufficient to expand lungs because of their underdeveloped, untrained state).
9	No apnea alarm because chest movements continue with inspiratory efforts.
10	Internal vacuum effect shunts systemic blood into intrathoracic organs, primarily the lungs. Capillaries rupture from high hydrostatic pressures, forming petechial hemorrhages on the linings of intrathoracic organs exposed to the negative pressures (Tardieu spots). This is potentially exacerbated by effective clamping of inferior vena cava and aorta at their hiatuses by the hypercontracted diaphragm.
11	Infant loses consciousness from rapid hypoxemia. RAM weaken.
12	Hypoxia, bradycardia and/or lack of respiratory movements finally trigger the alarm, but only 1–2 min remain before cardiac arrest.
13	Cyanotic, unresponsive child found by panicked parents who call 911 and initiate CPR. Chest compressions started. Rescue breaths attempted but met with airway resistance from improper neck positioning and the hypercontracted diaphragm. Parents not educated to open airway or look for chest rise.
14	Chest compressions resumed but the primary respiratory issue remains unaddressed.
15	Rescue breaths done hurriedly and again without confirmation. Panic and ineffective care continue.
16	Cardiac arrest.

CNS: central nervous system, DCC: diaphragmatic cramp-contracture, DD: diaphragm dysfunction (fatigue), RAM: respiratory accessory muscles, REM: rapid-eye-movement sleep, URI: upper respiratory infection. * Cold climate is a well-known SIDS risk factor (winter peaks in deaths). Indoor tobacco smoke will rise to the upper level of the home. This is typically where children’s bedrooms are located. Home heating would exacerbate this by concentrating smoke to the upper floor. Thus, home heating could be a major contributor to the climactic factor.

**Table 4:** Factors controlling respiratory outcome of nocturnal, unwitnessed seizures. It is important to recall the proposed sequence of events hypothesized here in SUDEP: seizure hyperstimulates the respiratory muscles, leading to diaphragm fatigue and neuromuscular excitability culminating in terminal DCC-apnea. But to trigger DCC, there must be a sudden increase in ventilatory workload. Depending on apnea duration and subsequent degree of hypoxemia, cardiac arrest and death could ensue if critical.

**Mortality factors in seizure**
Sz hyperstimulation of respiratory muscles (D ± RAM)
Sz hyperstimulation of bilateral D
Sz hyperstimulation of D is complete (all segments involved)
Critical preexisting diaphragm fatigue (male, prone, nicotine, dehydration, infections, myositis, pharyngeal OSA, pulmonary or neuromuscular diseases)
Ictal/postictal rebreathing of exhaled gases
Postictal inactivation of RAM by REM sleep or cramping
Complete DP from DCC
Acute R > L cardiac shunt from DCC precipitates critical hypoxemia (cardiac arrest)
**Survival factors in seizure**
Sz does not hyperstimulate respiratory muscles
Sz hyperstimulation of D only (not RAM, which can compensate)
Sz hyperstimulation of RAM only (not D, which can compensate)
Sz hyperstimulation of D is unilateral or partial
Subcritical sz duration re: metabolic derangements (lactate, electrolyte changes), overheating
Subcritical sz/postictal apnea duration re: hypoxemia (no cardiac arrest)
Postictal incomplete excitation of D (still functional) e.g., transient diaphragm spasms but not DCC

Sz: seizure, D: diaphragm, RAM: respiratory accessory muscles, DCC: diaphragm cramp-contracture, OSA: obstructive sleep apnea, DP: diaphragm paralysis; R: right, L: left.

**Table 5:** Proposed labs to reveal evidence of diaphragm fatigue/excitation, and potential markers.

Labs Tests (Screening, Trends and Respiratory Distress * PRN)
Nasopharyngeal swab (r/o respiratory viruses)
Hemoglobin (r/o anemia)
Extended electrolytes (sodium, potassium, calcium, magnesium, phosphate)
Lactate and VBG (pH, pCO_2_, HCO_3_)
CK-MM or sTnI (correlate with VBG and respiratory distress PRN)

PRN: Pro re nata (as necessary), r/o: rule out, VBG: venous blood gas, CK-MM: creatine kinase skeletal muscle isoenzyme, sTnI: fast isoform skeletal muscle troponin-I. * i.e., tachypnea, hypopneas, rib retractions, TAA, recurrent or prolonged apneas, desaturations, bradycardia, cyanosis, pallor, hypotonia, syncope, seizure, unexplained events, frequent hiccups, abdominal pulsations.

**Table 6:** Proposed monitoring and imaging to reveal evidence of diaphragm fatigue/excitation.

Investigations (Monitoring and Active Excitation PRN, Histology)
Continuous RIP and transcutaneous/esophageal D-EMG in high-risk individuals *
Bedside abdominal US and D-CEUS (perfusion) in suspected active DHD ^†^
Bedside fluoroscopy (“C-arm”) or dynamic chest radiography in active DHD
Diaphragm biopsy (myofiber disruptions, infiltrates, contraction bands, scars)

RIP: respiratory inductive plethysmography, D-EMG: diaphragm electromyography, TAA: thoracoabdominal asynchrony, US: ultrasound, D-CEUS: diaphragm contrast-enhanced ultrasound, DHD: diaphragm hyperexcitation disorder. * i.e., respiratory distress or lab abnormality (refer to Table 5), SIDS sibling(s). ^†^ Diaphragm spasms, myoclonus, flutter.

**Table 7:** Treatment of respiratory distress from diaphragm fatigue/excitation. Generalized interventions (left). Medications obtained upon literature review (right) are only preliminary findings, not corroborated. Some are active at both CNS and respiratory muscles.

General Interventions	Potential Medications
Supplemental oxygen	Caffeine, theophylline
Intravenous access	Chlorpromazine, haloperidol
Cardiac monitor, RIP, oxygen saturations, capnometry	SSRIs
CPAP, nasal mask or intubation/MV	Gabapentin, pregabalin
Minimize psychiatric stressors and pain	Carbamazepine, phenytoin, levetiracetam
Anti-seizure medications PRN	Benzodiazepines, amobarbital?
Correct hypovolemia and acidosis (bicarbonate?)	Cyclobenzaprine
Correct anemia and electrolyte disorders	β-agonists *
Stop antireflux medications	CACB ^†^
Treat infection (antibiotics, antivirals?)	ACE inhibitors
Treat bronchospasm (β-agonists, theophylline?)	Pentoxifylline
Body cooling measures PRN	N-acetylcysteine
Diaphragm or phrenic nerve pacing PRN	Erythropoietin
	Lidocaine?

RIP: respiratory inductive plethysmography, CPAP: continuous positive airway pressure; PRN: pro re nata (as necessary); SSRIs: selective serotonin reuptake inhibitors; CACB: calcium channel blockers; ACE: angiotensin-converting enzyme; * salbutamol, terbutaline, isoproterenol, procaterol; ^†^ nicardipine, verapamil, nifedipine.

## Data Availability

The original data presented in the study are openly available in ResearchGate at https://www.researchgate.net/publication/376356589_Diaphragm_Hyperexcitability_Disorders_Diaphragm_Flutter_and_Spasm_Case_Reports_Lit_Review_SPREADSHEET_by_Gebien (accessed on 9 August 2024).

## Supplementary Materials

The following supporting information can be downloaded at: https://www.mdpi.com/article/10.3390/diagnostics14202324/s1, Figure S1: Sleep polysomnograph in a preterm infant with silent squirming and respiratory instability (full image); Table S1: Differential diagnosis of case patient’s symptoms (bearhug pain apnea); Table S2: Why diaphragm cramp is unknown to medicine; Patient’s Perspective; Video S1: Rescue breath technique, available at https://youtu.be/3lWcsamOYOw (accessed on 9 August 2024).

## Abbreviations

CBN: contraction band necrosis, CK: creatine kinase, CK-MM: creatine kinase skeletal muscle isoenzyme, CPAP: continuous positive airway pressure, CPK: creatine phosphokinase, D-EMG: diaphragm electromyography, DCC: diaphragm cramp-contracture, DD: diaphragm dysfunction (fatigue or insufficiency), DP: diaphragm paralysis, Edi: electrical activity of diaphragm, HE: hypoxemic episode, HHVD: hypoxemic/hypercapnic ventilatory depression, IAM: influenza-associated myositis, ICM: intercostal muscles, IVC: inferior vena cava, MV: mechanical ventilation, OSA: obstructive sleep apnea, PH: pulmonary hypertension, RAM: respiratory accessory muscles, RIP: respiratory inductive plethysmography, RSV: respiratory syncytial virus, SIDS: sudden infant death syndrome, STnI: skeletal muscle troponin-I, SUDC: sudden unexplained death in childhood, SUDEP: sudden, unexpected death in epilepsy.

## Appendix A

**Table A1:** Acute and chronic medical conditions that can present emergently with respiratory decompensation by critical diaphragm fatigue. Upon literature review, many fatalities had exhibited terminal apnea prior to cardiac arrest. This could have occurred by central or peripheral causes. However, only the latter was demonstrated in experimental septic, cardiogenic and hypovolemic shock, from “fatigue-induced failure of the [inspiratory] contractile machinery” (consistent with the DCC mechanism). Tailoring therapies that reduce ventilatory muscle fatigue might improve survival.

Condition (All Ages)	Citation
**Neurologic & myopathic**	
Neuropathy (e.g., ALS, GBS, myasthenia gravis)	[46]
Myopathy (e.g., MS, muscular dystrophy, polymyositis)	[176]
Critical-illness polyneuropathy	[29,96]
Generalized tonic-clonic and focal seizures	[92,115]
**Lower and upper airway**	
Asthma, COPD, pneumonia	[29,96,177]
Bronchiolitis	[167]
Croup, acute epiglottitis, bacterial tracheitis	[178]
Acute lung injury, ARDS	[179]
Cystic fibrosis	[180]
**Infection**	
Influenza and other viral URI	[89,181]
SARS-CoV-2 (COVID-19)	[47,149]
Severe gastroenteritis dehydration	[86,111]
Rabies and tetanus, Lyme disease, polio	[29]
**Shock**	
Septic	[74,95]
Hypovolemic	[108,182]
Hemorrhagic	[114]
Cardiogenic (e.g., AMI, arrhythmias, PE, tamponade)	[108,131]
Neurogenic and noncardiogenic pulmonary edema	[183]
**Metabolic acidosis**	
DKA, lactic acidosis, starvation ketosis	[25,45]
Toxicities (e.g., metformin, methanol, ethanol, ethylene glycol)	[184]
**Medications and neurotoxins**	
Malignant hyperthermia	[28,185]
Succinylcholine and anesthesia induction	[38]
Nicotine, botulism, tetanus, curare	[31,37]
Envenomations, neurotoxins	[37,186]
Aminoglycoside antibiotics	[187]
**Endocrine & electrolyte disorders**	
Pheochromocytoma, catecholamine storm	[40]
Thyroid, parathyroid hormones	[188]
Corticosteroids and aldosterone	[188]
Diabetes, hyperglycemia	[26,188]
Hyperkalemia (and extreme acidosis)	[43]
Hypokalemia	[44]
Hypomagnesemia	[41,129]
Hypophosphatemia	[142]
Hypocalcemia	[42]
Hypercalcemia	[189]
Hyponatremia	[190]
Hypernatremia	[191]
**Trauma**	
Phrenic nerve injuries: birth trauma, soft tissue injuries	[55,56]
Diaphragmatic injuries: rupture, laceration, contusion, paralysis	[62,192]
Cervical spinal cord injuries	[50]
Severe winding injuries, traumatic cardiac arrests	[64,193]
**Exposures**	
Electrocutions	[23,24,194]
Mechanical asphyxiation (e.g., restraint cardiac arrest, crush syndrome)	[67,68]
Heat stroke/hyperthermia (e.g., vehicular, marathon runner arrests)	[73,154,195,196,197]
Chemical asphyxiants, inhalational injuries	[69,198]
Cold water immersion and near-drownings	[70]
Barotrauma	[199]
**Other**	
Malnourishment	[45]

ALS: amyotrophic lateral sclerosis, GBS: Guillain-Barré syndrome, MS: multiple sclerosis, ARDS: acute respiratory distress syndrome, AMI: acute myocardial infarction, PE: pulmonary embolism.
