# Supplementary material for: Uncovering Diaphragm Cramp in SIDS and Other Sudden Unexpected Deaths
Source: Diagnostics (Basel). 2024 Oct 18;14(20):2324. doi: 10.3390/diagnostics14202324 (PMC11506607; doi:10.3390/diagnostics14202324)
Supplement: Supplementary file 1 [file diagnostics-14-02324-s001.zip › DCC GEBIEN Supplemental Material.pdf]

SUPPLEMENTAL MATERIALS

"Uncovering Diaphragm Cramp in SIDS and other Sudden Unexpected Deaths"

Dov Gebien MD and Michael Eisenhut MD

October 10, 2024 (v.3)

Figure S1

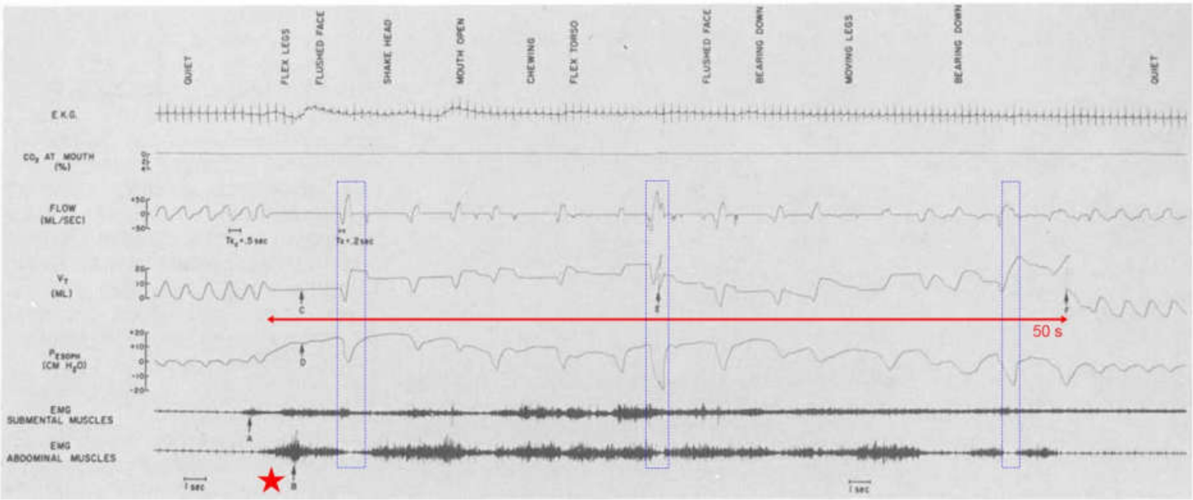

Figure S1. Sleep polysomnograph in a preterm infant with silent squirming and respiratory instability (full image). In addition to tidal volume (V_T) and airflow, important parameters include esophageal pressure (P_{ESOPH}) and abdominal muscle EMG (EMG_{ABDO}). Oxygen saturations not available. An abnormal sequence begins at "A-B" (red star), where EMG_{ABDO} activity was followed by a slow rise in P_{ESOPH} ("D"), both of which remained abnormally elevated for 50 s until the end of the episode. Simultaneously, normal tidal breathing was interrupted at "C" by a 4 s apnea, associated with silent squirming (flexing legs, flushing, etc.). This was followed by a stepwise, breath-to-breath increase in end-expiratory volume consistent with breath stacking (occurred twice and required downshifting at "E" and "F" to stay on scale). Unusually, these respirations (seen in V_T and Flow) were reversed in which *expiration preceded inspiration* (not discussed by authors). Respirations were also slowed (bradypnea) and of lower volumes (hypopneas). Despite elevated EMG_{ABDO} and P_{ESOPH}, negative deflections in the latter (and hypopneas) likely reflect a composite of inspiratory work by the diaphragm and accessory muscles mediated by neural breathing (but not abdominal muscles as they are expiratory). When short, <1 s, pauses in EMG_{ABDO} occurred (blue boxes), unusually large, hyperpneic breaths were taken, perhaps compensation to developing hypoxia and hypercapnia. When EMG_{ABDO} finally abated ("F"), normal tidal breathing resumed. EMG_{ABDO} was interpreted to be from abdominal muscle activity, perhaps by intentional "Valsalvas" (to blow off excess air from hyperinflation). Alternatively, it could reflect cross contamination by diaphragm contractions given this was measured transcutaneously. Either muscle group could elevate P_{ESOPH}. In other words, diaphragm spasms could have caused the silent squirming episode with its hypoventiliations, breath stacking, hyperinflation and reversed respirations. Finally, the latter is reminiscent of the case patient's rescue breath technique. Perhaps the reversal was intentional. Reprinted with permission of the American Thoracic Society. Copyright © 2024 American Thoracic Society. All rights reserved. Cite: Abu-Osba YK, et al. 1982. Breathing pattern and transcutaneous oxygen tension during motor activity in preterm infants. *Am Rev Respir Dis*. Apr;125(4):382-7. pp382-387 [33]. The American Journal of Respiratory and Critical Care Medicine (previously known as The American Review of Respiratory Disease) is an official journal of the American Thoracic Society.

Supplemental Tables

Table S1. Differential diagnosis of case patient's symptoms (bearhug pain apnea). Various proposed etiologies of pediatric rib pain and apnea are listed separately (A and B, respectively), and combined (C). Conditions for inclusion in (C), as suggested by the patient's report, were recurrent nocturnal spontaneous sudden onset cramp-like bilateral rib pain with simultaneous inspiratory arrest. Clinical reasoning yielded six final diagnoses with relative degrees of clinical confidence.

**A. Unilateral and Bilateral Pediatric Rib Pain***		**Apnea?**	**Recurrent?†**
	Rib fracture, muscle strain, intercostal neuralgia	No	Possible
	Fibromyalgia, juvenile rheumatoid arthritis	No	Possible
	Pleurisy, pleurodynia	No	Possible
	Tumours of chest wall and ribs	No	Unlikely
	Pneumothorax, pneumomediastinum	No	Unlikely
	Electrical injury	**Possible**	**Possible**
	Panic attack, somatoform and fictitious disorders, malingering	**Possible**	**Possible**
	Child abuse	**Possible**	**Possible**
	Intercostal muscle cramp(s)	**Possible**	**Possible**
	Diaphragm cramp-contracture	**Possible**	**Possible**

**B. Pediatric Apnea***		**Rib Pain?**	**Recurrent?†**
Mechanical	Obstructive sleep apnea	No	Yes
	Upper airway trauma, burns, foreign body	No	Unlikely
	Airway tumour, polyps, bilateral vocal cord paralysis	No	Unlikely
	Tonsillar hypertrophy, tracheal webs & atresia, macroglossia	No	Unlikely
	Epiglottitis, abscess, croup	No	Unlikely
	Anaphylaxis	No	Possible
	Intercostal muscle cramp	**Yes**	**Possible**
	Diaphragm cramp-contracture	**Yes**	**Possible**
Nervous system	Seizure	Unlikely	Yes
	Cardiac arrhythmia	No	Possible
	Medications (opioids, neuromuscular blockers)	No	Unlikely
	Toxins (botulism, tetanus, curare, tetrodotoxin)	No	Unlikely
	Exposures (carbon monoxide, cigarette smoke)	No	Possible
	Idiopathic central sleep apnea, periodic breathing, Cheyne-Stokes, obesity hypoventilation syndrome	No	Yes
	Parasomnias (sleep paralysis, night terrors)	No	Possible
	Breath-holding	No	Possible
	Panic attack, somatoform and fictitious disorders, malingering	**Possible**	**Possible**
	Child abuse	**Possible**	**Possible**
Mixed	Acid reflux with laryngospasm	No	Yes
	Upper and lower respiratory infections	No	Yes
	Aspiration pneumonia	No	Possible

	Sepsis and serious bacterial infections	No	Possible
Traumatic	Head trauma, Raised intracranial pressure	No	Unlikely
	Spinal cord injury, bilateral phrenic nerve injuries	Possible	Unlikely
	Bilateral pneumothoraces, pneumomediastinum	Yes	Unlikely
	Electrical injuries	**Possible**	**Possible**
	Diaphragmatic spasm from winding injury	**Possible**	Unlikely

**C. Recurrent Bilateral Rib Pain and Apnea**[*†]		**Clinical Confidence**
	Repeated electrical injuries	Low
	Recurrent seizures	Medium
	Panic attack, somatoform and fictitious disorders, malingering	Medium
	Child abuse	Medium
	Bilateral intercostal muscle cramps	High
	Bilateral diaphragm cramp-contracture	High

[*]List is inexhaustive. [†]"Recurrent" refers to relapsing and remitting. Bold: higher clinical suspicion, Italics: putative (unproven).

Table S2. Why diaphragm cramp is unknown to medicine. Speculation is provided as to how DCC has evaded detection historically.

	**Spontaneous DCC** (s-DCC): Is a proposed mechanism of spontaneous respiratory arrests in individuals with critical diaphragm fatigue (causing some cases of SIDS, SUDC and SCD). Sleep is an especially vulnerable time, primarily because of REM sleep inactivation of airway dilator and respiratory accessory muscles (suddenly adding diaphragmatic workload). The process is rapid, with only 5-10 s before hypoxic syncope and 1-2 min before cardiac arrest ensue (however, can be aborted by rescue breaths).<br>**Traumatic DCC** (t-DCC): Is thought to be a severe form of abdominal winding injury (celiac or solar plexus syndrome). It occurs from a heavy, non-penetrating blow to the epigastrium or chest, stunning the diaphragm and inducing respiratory arrest by a *sustained* diaphragmatic spasm (diaphragm cramp). As above, this would rapidly progress to syncope, cardiac arrest and death if not aborted. In contrast, a milder impact induces forced apnea from a *transient* diaphragm spasm, wherein the victim momentarily cannot inspire until the diaphragm recovers.<br>**Seizure DCC** (sz-DCC): Seizure activity is proposed to hyperstimulate the diaphragm via the phrenic nerves (causing SUDEP by terminal apnea). This presents as a mixture of periictal hyperpneas, hypopneas and apneas, causing net hypoxemia (and sometimes cyanosis). This alone can trigger DCC. However, if seizure continues, lactic acidosis and critical hypoxemia develop (both of which impair contractility), and could also catalyze DCC. Additionally, even a postictal roll to prone position or onset of REM sleep can trigger DCC (both suddenly add diaphragmatic workload).
1	Historically, compared to the other vital pump (heart), the diaphragm has been grossly understudied and underappreciated as causing serious disease.
2	DCC is thought to have exceptionally high mortality (few survivors who live to talk about it). The most commonly affected age groups in s-DCC are infants and young, preverbal children (childhood amnesia). Also, nocturnal cases are triggered in REM sleep, a deep sleep stage that makes later recall of events less clear. Those who survive hypoxic syncope of t-DCC will likely have retrograde amnesia, unable to recall the respiratory arrest (e.g. collapse of NFL player in 2023).
3	The process is unwitnessed in most nocturnal cases, and silent because of the inspiratory arrest (victim unable to cry out for help).
4	Death from DCC respiratory arrest is rapid and mimics other conditions, like choking, seizure and collapse from a sudden cardiac arrest (e.g. VFib, VTach). This would lead to misclassification of the primary cause of death. Those in fatal winding injuries would be classified as traumatic cardiac arrests or commotio cordis.
5	Diaphragm spasms and cramps are not visible because the diaphragm is internal. Specialized studies are needed to detect; however, they are spontaneous, unpredictable and transient. Diaphragm EMG currently offers the best alternative (to monitor continuously), however, spasms and cramps mimic artifacts from electrical "noise" and body movements.
6	Similar to VFib and VTach, pathological excitation of pump muscles do not persist postmortem (undetectable at autopsy). Also, the internal mechanical airway obstruction of DCC is not visible (as the offending agent is the diaphragm itself).
7	Standard autopsies in sudden unexpected deaths generally inspect the diaphragm grossly but omit histology (missing the myopathic changes of DCC). No gross abnormalities are detectable.
8	In non-monitored inpatients, critical apnea is silent and, unfortunately, avoids detection. Even in those receiving respiratory monitoring, airflow is not measured; rather chest impedance (respiratory movements). Because of continued chest movements in DCC (attempting to breathe against obstruction), the apnea alarm is

not triggered. Also, where many false alarms occur, as is common on busy wards, apnea alarms do not receive immediate attention (as does cardiac arrest). Therefore, it is plausible the *apnea of DCC is being missed in both settings*. Lastly, oxygen desaturation alarms are a late finding, missing the critical event.

DCC: diaphragm cramp-contracture, SIDS: sudden infant death syndrome, SUDC: sudden unexplained death in childhood, SCD: sudden cardiac death, SUDEP: sudden unexpected death in epilepsy, EMG: electromyography, VFib: ventricular fibrillation, VTach: ventricular tachycardia

Patient's Perspective

This account was written by the case patient, who is a practicing medical doctor born in 1970 (52 years old at disclosure). With trauma counselling in 2022, he explored life-threatening breathing emergencies that had awakened him from sleep sporadically throughout his childhood and youth. He feels he came within a breath of losing his life each time. It appears his memories were repressed as a defense (survival) mechanism. Notably, he had multiple childhood risk factors overlapping with those identified in SIDS.

"One night while alone in bed at 7 or 8 years old, I suddenly awoke from a sharp and excruciating pain in my ribs that felt like someone had picked me up from behind in a tight bearhug. The constant-intensity pain radiated from back-to-front in a C-shaped distribution, with sternal sparing. I couldn't breathe in at all and was also baffled because my mouth had opened involuntarily when I first gasped for air. This all happened in under two seconds. As I fully awoke, turning to tell "the person who was bear-hugging me to stop", I was shocked to realize I was in my bed and that nobody was there. The bearhug and inability to inhale (inspiratory apnea) persisted.

Despite the impending sense of doom I began troubleshooting by experimentation. But when I tried inhaling more forcefully it was met with equal and opposite, complete resistance to airflow. It was futile. My next test, to exhale, was successful though and I remember telling myself not to lose all the air in my lungs so to conserve it. I still couldn't breathe in, and the pain continued. I was not panicked and did not have stridor, choking, fullness or a foreign body sensation in my throat.

What I did next was lifesaving.

I tried something new by partially exhaling followed immediately by three short-burst inhalations with pursed lips to increase inspiratory pressure [see Video S1 for more]. To my relief, the pain and apnea resolved immediately, and normal breathing resumed. Crisis averted, so I went back to sleep *as only a seven-year-old can despite a near-death experience*. Soon afterwards, I noticed a pilot on TV spinning in a centrifuge using the same distinctive pursed-lip breathing technique. I had never seen that before.

The following morning I was puzzled because the pain had all but disappeared. I had already learned from ankle sprains that severe pain like that typically lasted days if not longer.

The breathing emergencies recurred sporadically throughout my childhood and youth but only at night while fast asleep. I do not know if I had any associated illnesses, however, I did have a fair bit of diarrhea throughout my childhood and youth of unknown cause.

Eventually I recognized, in my sleep, prodromal flickering pains in my ribs (fasciculations) to be a warning sign of the impending bearhug apnea like that of the first episode. I would wake from this and take in a quick breath to prevent the "big" pain from kicking in. I can say with absolute certainty, the pain was a muscle cramp. I should also note the bearhug came on just at the *very end of expiration* (if not averted by quickly breathing in). I can still recall how the pain always spread from a spot in my right posterolateral ribs to the encircling bearhug in a matter of milliseconds upon the next breath.

The rescue breaths were so loud and high-pitched, I remember being awakened one night by the sound. It appears I had grown so accustomed, that *I did them in my sleep*. Oddly, I also recall telling myself to keep it quieter the next time because I didn't want to wake anybody else up. My memory of that bedroom places it in our family's newer home, therefore, between ages 17 and 23. I'm not certain if I had any further episodes after that.

There are a few notable childhood medical conditions to share.

Due to severe gastroesophageal reflux, malnourishment and failure to thrive over my first year of life I underwent an uncomplicated open exploratory-laparotomy with Nissen fundoplication at 18-

months of age. It definitively treated a congenital hiatal hernia. I recovered well, quickly gained weight and do not recall having reflux symptoms as I grew older.

Since age 8 or 9 years, I frequently experience painful fasciculations and muscle cramps that cause contracture-like stiffness in the affected limbs. One day the small muscles of the hand are affected (claw hand), whereas the next involves larger ones such as a calf or posterior thigh. With repeated episodes over the years, fasciculations alerted me to prevent imminent cramps by quickly stretching the affected muscle. I have not received a diagnosis for this ongoing condition.

In addition, beginning at roughly 10 years old, I occasionally became suddenly and extremely fatigued during prolonged, intense exercise. I learned it occurred when not eating properly beforehand. Carbohydrate-rich foods prevented and aborted symptoms. This condition was undiagnosed at the time of writing but is consistent with hypoglycemia from McArdle's (glycogen storage) disease.

Social history: I was the second male child of a Gravida 4, Para 2 smoker. Brother denied sleep-related pain, breathing issues or reflux. I slept alone in an *upstairs* bedroom in a household containing *cigarette smoke* that was *heated* in wintertime.

Notably, I had stopped thumb-sucking around the same time as onset of the breathing emergencies (important because pacifiers are SIDS protective). I cannot think of anything else that had changed which could explain why this all started at age 7 and not sooner (how fortunate I am)!

In terms of childhood risk factors overlapping with SIDS, mine were numerous including male sex, reflux, chronic diarrhea, residing in a colder climate, household cigarette smoke from maternal use, nocturnal diaphoresis, deep sleeping with preference for the prone position and tendency to pull bed linens over my shoulders and head.

I do not have a history of panic attacks, anxiety, depression or sleep disorders such as obstructive sleep apnea, night terrors or sleep paralysis. No cardiac abnormalities such as palpitations, exercise intolerance or syncope. No respiratory issues such as bronchospasm, pneumonia, choking episodes or prolonged cough or colds. No allergies, anaphylaxis or unusual childhood infections. No seizures, atypical headaches or focal muscle weakness. No family history of cardiac arrhythmias or sudden unexpected deaths, including SIDS."

* * *

I may have survived these life-threatening events because, as opposed to an infant, I had the benefit of wherewithal and coordination possessed by an older child. It is only now upon reflection as an adult do I realize how lucky I am to be alive. I am determined to eradicate DCC.

Sleeping children need our help.

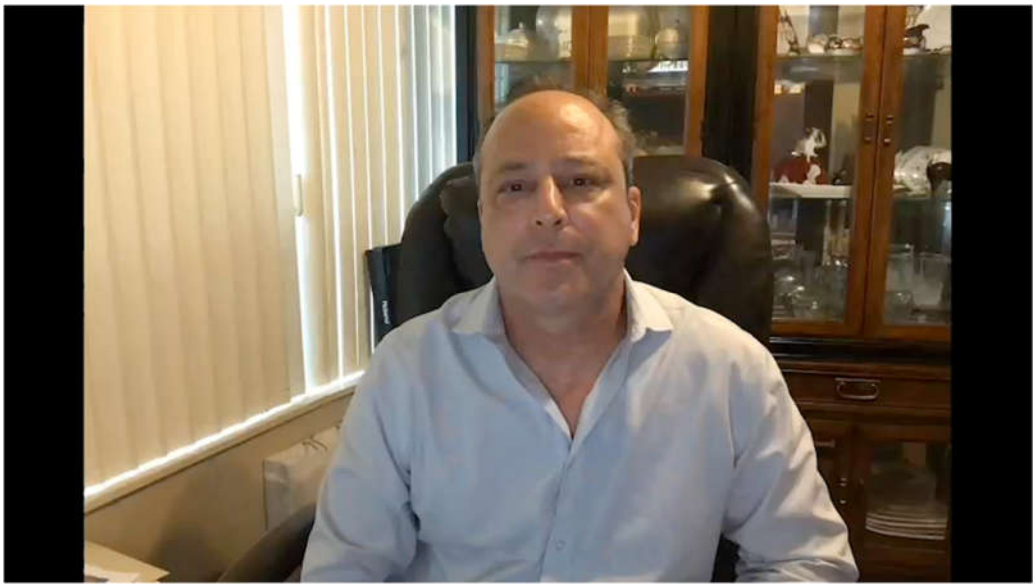

Video S1. Rescue breath technique. Dr. Gebien demonstrates the patient's rescue breath technique by exhaling followed by three short-burst, positive-pressure inspirations using his tongue to completely occlude the airway by percussing the hard palate. The case patient recounted how this resembled "a pilot breathing in a centrifuge" (prevents compression of the lungs under centripetal forces).
